# Multi-Method Molecular Characterisation of Human Dust-Mite-associated Allergic Asthma

**DOI:** 10.1038/s41598-019-45257-1

**Published:** 2019-06-20

**Authors:** E. Whittle, M. O. Leonard, T. W. Gant, D. P Tonge

**Affiliations:** 1 School of Life Sciences, Faculty of Natural Sciences, Keele University, ST5 5BG Newcastle, England; 2 Centre for Radiation, Chemical and Environmental Hazards, Public Health, OX11 0RQ Didcot, England

**Keywords:** Gene expression analysis, Transcriptomics

## Abstract

Asthma is a chronic inflammatory disorder of the airways. Disease presentation varies greatly in terms of cause, development, severity, and response to medication, and thus the condition has been subdivided into a number of asthma phenotypes. There is still an unmet need for the identification of phenotype-specific markers and accompanying molecular tools that facilitate the classification of asthma phenotype. To this end, we utilised a range of molecular tools to characterise a well-defined group of female adults with poorly controlled atopic asthma associated with house dust mite (HDM) allergy, relative to non-asthmatic control subjects. Circulating messenger RNA (mRNA) and microRNA (miRNA) were sequenced and quantified, and a differential expression analysis of the two RNA populations performed to determine how gene expression and regulation varied in the disease state. Further, a number of circulating proteins *(IL-4*, *5*, *10*, *13*, *17* *A*, *Eotaxin*, *GM-CSF*, *IFNy*, *MCP-1*, *TARC*, *TNFα*, *Total IgE*, *and Endotoxin)* were quantified to determine whether the protein profiles differed significantly dependent on disease state. Finally, we utilised a previously published assessment of the circulating “blood microbiome” performed using 16S rRNA amplification and sequencing. Asthmatic subjects displayed a range of significant alterations to circulating gene expression and regulation, relative to healthy control subjects, that may influence systemic immune activity. Notably, several circulating mRNAs were detected in just the asthma group or just in the control group, and many more were observed to be expressed at significantly different levels in the asthma group compared to the control group. Proteomic analysis revealed increased levels of inflammatory proteins within the serum, and decreased levels of the bacterial endotoxin protein in the asthmatic state. Comparison of blood microbiome composition revealed a significant increase in the Firmicutes phylum with asthma that was associated with a concomitant reduction in the Proteobacteria phylum. This study provides a valuable insight into the systemic changes evident in the HDM-associated asthma, identifies a range of molecules that are present in the circulation in a condition-specific manner (with clear biomarker potential), and highlights a range of hypotheses for further study.

## Introduction

Asthma is a chronic inflammatory disorder of the airways and is a global public health concern due to increasing prevalence and mortality rates^1–4^. The World Health Organisation has estimated that 300 million people are living with asthma, and that 250,000 individuals die prematurely each year as a result of the disease^5^.

Asthma can develop during childhood (early-onset) or in adulthood (late-onset) and is characterised by chronic inflammation of the airways and intermittent episodes of reversible airway obstruction^6,7^. Over time, chronic inflammation of the airways results in airway hyper-responsiveness and structural changes, including airway fibrosis, goblet cell hyperplasia, increased smooth muscle mass, and increased angiogenesis^7,8^.

The causes of asthma are multifactorial, and include a complex variety of environmental, immunological, and host genetic factors^7,9–13^. Disease typically occurs in genetically predisposed individuals^13,14^, and clinical presentation is highly heterogenous^15^. Disease can vary greatly in terms of disease onset and response to treatment^16^. It can present as a chronic, stable disease, but also as intermittent asthma exacerbations that can be fatal^17^. Symptoms can be mild or severe and arise as a result of a multitude of factors, including immunoglobulin-E (IgE) mediated allergic responses, exposure to pollutants, exercise, stress, or airway infections^17^.

The complex nature of asthma pathogenesis has resulted in speculation as to whether asthma is a single disease, or a spectrum of related diseases with subtle but distinct differences in aetiology and pathophysiology^18,19^. This has led to asthma being separated into a number of phenotypes, which are then further subdivided into several endotypes^6,15,18–20^. These asthma phenotypes are triggered by complex gene-environment interactions and respond differently to the various asthma medications available. Individuals with eosinophilic asthma, for instance, have been reported to have a good therapeutic response to inhaled or oral corticosteroid therapy, whereas individuals with neutrophilic asthma have been found to respond poorly to this therapeutic approach^21^.

Diagnostic tools for identifying the various asthma phenotypes are limited, and thus optimal treatment protocols are not being utilised in a number of patients. Moreover, despite decades of research, there has been little progress in the development of treatments since the introduction of inhaled ß2 adrenoceptor 2 selective agonists (1969) and inhaled glucocorticosteroids (1974)^15^. Long-term use of these medications has been associated with a number of health concerns^22^, including the stunting of growth in children^23^, cataract development^24,25^, osteoporosis^26,27^, and cardiovascular events^28^. Overall, an estimated 5–10% of asthmatics fail to respond to conventional medications^29^. In order to improve patient response to treatment, and/or assist in the development of new therapeutics, an improved knowledge of the molecular mechanisms that underlie the various asthma phenotypes is required. Long-term, this may also facilitate the targeted use of conventional asthma therapies, and facilitate the development of new medications aligned to the individual asthmatic phenotypes, subsequently reducing asthma mortality and improving quality of life.

The focus of this study was to characterise, at the molecular level, a small but well-defined cohort of female patients with atopic asthma associated with house dust mite (HDM) allergy. Atopic asthma prevalence during childhood is typically higher in males compared to females^30–33^. However, when the disease does develop in females it is more likely to persist into adulthood^34,35^, be associated with additional atopic complications, such as allergic rhinitis and atopic dermatitis^36,37^, and increased susceptibility to asthma exacerbations^36,38–40^. Females are also more prone to developing severe asthma^35,41^, suffer asthma control problems^36^, and reduced quality of life^36^ that is associated with higher asthma mortality rates^41,42^.To increase our understanding of atopic asthma in adult females is therefore crucial. To this end we performed a comprehensive molecular characterisation of (1) circulating mRNAs, (2) circulating microRNAs, (3) circulating protein-based markers of the immune response and (4) integrated these data with our previous work characterising evidence of a circulating microbiome.

## Methods

### Donor population

Female atopic asthmatic individuals (n = 5) with physician-diagnosed allergy to House Dust Mite antigen, and gender and age-matched healthy control subjects (n = 5) were recruited to the study via SeraLabs Limited. Asthma patients were selected on the basis that they had developed atopic asthma during early childhood and that their condition had continued into adulthood and remained “poorly controlled”. A full list of recruitment criteria is presented in **(**Table 1**)**.

**Table 1:** Donor population characteristics required for the study.

**Patient Criteria**
• Have a BMI <30
• Be a non-smoker
• Have been diagnosed with atopic asthma during childhood
• Have severe/poorly controlled asthma
• Must not be on any oral steroid treatment
• Must be allergic to the house dust mite antigen
• Must not have diabetes, COPD, or hypertension

Whole blood was drawn, following alcohol cleansing of the skin surface, into EDTA containing tubes and stored on ice prior to centrifugation at 1000 × *g* to obtain the plasma component. All samples were analysed anonymously, and the authors obtained ethical approval and written informed consent to utilise the samples for the research reported herein. The Independent Investigational Review Board Inc. ethically approved sample collection by Sera Laboratories Limited from human donors giving informed written consent. Furthermore, the authors obtained ethical approval from Keele University Ethical Review Panel 3 for the study reported herein. All methods were performed in accordance with relevant guidelines and regulations.

### Analysis of inflammatory proteins

Plasma levels of interleukin (IL)-4, IL-5, IL-10, IL-13, IL-17A, IFNy, TARC, Eotaxin, GM-CSF, MCP-1, RANTES, and TNFα, were determined using a qualitative enzyme-linked immunosorbent assay (ELISA) custom designed for this study. Two multi-analyte sandwich ELISAs (Qiagen) were used, and analysis of the inflammatory proteins was achieved using the recommended Multi-analyte ELISArray kit protocol (QIAGEN). Given the qualitative nature of this kit, results were expressed as the optical density at 450 nm, with greater OD values indicating higher levels of the analyte in question, as recommended by the manufacturers directions. Statistical analysis was performed by carrying out a Shapiro-Wilk normality test and a Wilcox rank sum test using *R* software Version 3.5.0.

### Quantitative analysis of total IgE

Total plasma immunoglobulin E (IgE) was determined using sandwich ELISA (Genesis Diagnostics Ltd). Determination was performed in duplicate using the recommended protocol, with absorbance measured at 450 nm using an ELX800 spectrophotometer (BioTek). Statistical analysis was performed by carrying out a Shapiro-Wilk normality test and an unpaired T test using *R* software Version 3.5.0.

### Quantitative analysis of endotoxin concentration

Circulating bacterial endotoxin concentration was measured using the Pierce^TM^ Limulus Amebocyte Lysate (LAL) Chromogenic Endotoxin quantitative kit (Thermo Scientific). The assay was performed in triplicate using the recommended protocol, with absorbance measured at 450 nm using an ELX800 spectrophotometer (BioTek). Statistical analysis was performed by carrying out a Shapiro-Wilk normality test and an unpaired T test using *R* software Version 3.5.0.

### Total RNA extraction

Total RNA was extracted from 500 µl of human plasma using the Qiagen serum and plasma miRNeasy kit. The quantity and quality of the RNA extracts was determined using the QuBit fluorimeter (Invitrogen) and BioAnalyzer (Agilent).

### Library preparation and next generation sequencing

Messenger RNA (mRNA) sequencing libraries were prepared using the SMARTer Universal Low Input RNA kit, and sequenced (Illumina HiSeq 2000) with a paired-end 90 nucleotide read metric. Small RNA sequencing libraries were prepared using the TruSeq small RNA library kit (Illumina), and sequencing was conducted on the Illumina HiSeq 2000 platform. Raw sequencing data were trimmed of sequencing adaptors and low-quality reads removed using the “Trim Galore” package – a wrapper that incorporates CutAdapt and FastQC. For whole transcriptome analysis, quality-controlled reads were aligned to the Human Genome build hg19 using TopHat, a splice-junction aware mapping utility necessary for the successful mapping of intron-spanning (multi-exon) transcripts. Transcriptome assembly was performed using CuffLinks and a merged transcript representation of all samples produced using CuffMerge. Transcripts expressed at significantly different levels between the asthma and control samples were identified using CuffDiff, with a Q value ≤ 0.05 considered significant^43^. MicroRNA (miRNA) analysis was performed by mapping miRNA reads to miRbase Version 21 using sRNAtoolbox^44^. Differential expression of the miRNA reads was determined following statistical analysis with edgeR for R^45^.

### Biological pathway analysis

The biological impact of mRNA and miRNAs that were differentially expressed between asthma and control subjects (defined as Q ≤ 0.05 in the mRNA dataset; and FDR ≤ 0.05 in the miRNA dataset) were investigated using a number of techniques;

1. The likely impact of individual differentially expressed genes on asthma pathogenesis was analysed by comparing each gene to a recently released database of genes associated with asthma pathology (AllerGAtlas, 2018). A literature search was then utilised to determine the effects of the identified genes on asthma pathology and immune function.
2. Functional analysis was performed on the differentially expressed miRNA using DIANA-miRPath V3.0. Biological pathways likely to be affected by the differentially expressed miRNAs were determined using *in silico* predicted targets from TargetScan v6.2.
3. The combined effects of differential gene and miRNA expression on known biological processes was then explored using Ingenuity Pathway analysis (IPA) software. Causal inference analysis was applied to determine upstream regulators that may explain the pattern of differential expression seen. Causal inference analysis involved the generation of an enrichment score (Fisher’s exact test *P* value) and a Z score to determine the possible upstream biological causes of the differential gene expression observed in the asthmatic subjects^46^. The enrichment score measured the overlap of observed and predicted regulated gene sets, whilst the Z score assessed the match of observed and predicted up/ down regulation patterns^46^. Putative regulators that scored an overlap P value ≤ 0.05 were deemed statistically significant, and the Z scores were used to determine the activity of the putative regulators (an upstream regulator with a Z score greater than 2.0 was considered activated, whilst an upstream regulator with a Z score less than −2.0 was considered deactivated). Causal inference analysis was also used to predict the downstream effects the differentially expressed genes and miRNA could have on biological processes and functions in the asthmatic subjects.

### Circulating microbiome analysis

We have previously reported evidence of a circulating microbiome in the blood of both asthmatic and healthy patients^47^ using oligonucleotide primers reported in (Supplementary Materials, S1). Here, we re-analysed this existing data^47^ with the aim of identifying organisms that were differentially present or abundant dependent on disease status. The QIIME pipeline was used for quality filtering of DNA sequences, demultiplexing, and taxonomic assignment. Alpha diversity was determined by calculating Shannon and Chao1 diversity indices. Differences in relative abundance were calculated by performing Shapiro-Wilk normality tests and the appropriate statistical test (unpaired T tests when the samples displayed Gaussian distribution and Wilcox rank sum test when the samples did not display Gaussian distribution) on bacterial abundance data (read counts normalised to the total number of bacterial reads per patient) using *R* software Version 3.5.0.

In addition to standard statistical tests, the linear discriminant analysis effect size (LefSe) method was used to identify the bacterial taxa most likely to explain the differences in microbial populations present in the asthmatic cohort compared to the control cohort. In brief, the non-parametric factorial Kruskal-Wallis sum-rank test was applied to the 16S relative abundance data in order to detect features with significant differential abundance in the asthmatic cohort compared to the control group. A set of pairwise tests among subclasses using the unpaired Wilcoxon rank-sum test were then carried out to assess whether the detected differences in relative abundance were consistent with respect to biological behaviour. Linear discriminant analysis (LDA) was then performed to predict the effect of each identified differentially abundant bacterial taxa.

## Results

### Patient recruitment and characterisation

Five female asthmatic subjects were recruited in accordance with the inclusion criteria detailed in (Table 1). The mean age of the asthmatic subjects was 39.6 ± 11.7 years, and all had been clinically diagnosed with atopic asthma during early childhood (mean age of onset = 6.2 ± 3.2 years) (Table 2). At the time of sample collection, the asthmatic subjects were on prophylactic therapy to minimise the occurrence of disease symptoms (see Supplemental Material, S2). Asthma severity was determined using the internationally recognised Asthma Control Questionnaire (ACQ)^48,49^, and all the asthmatic subjects scored a total ≥ 10.0 (mean total score = 10.8 ± 0.75) (see Supplemental Material, S2). Additionally, three of the asthmatic subjects were clinically diagnosed with other atopic diseases, including allergic rhinitis, allergic dermatitis, and nasal polyps (see Supplemental Material, S2).

**Table 2:** Characterisation of the asthmatic (n = 5) and control subjects (n = 5) at the time of sample collection. S.D. = standard deviation.

Characteristic	Allergic Asthmatics	Non-Asthmatics
**Demographic characteristics**		
**Age - yr**		
Mean (S.D)	39.6 (11.7)	39.4 (10.3)
Range	19–52	23–49
**Race or ethnic group – no. (%)**		
Caucasian	2 (40)	2 (40)
Hispanic	3 (60)	3 (60)
**Sex – no. (%)**		
Female	5 (100)	5 (100)
Male	0 (0)	0 (0)
**Smoking History**		
**Smoking Status – no (%)**		
Never Smoked	5 (100)	5 (100)
Former Smoker	0 (0)	0 (0)
Smoker	0 (0)	0 (0)
**BMI**		
Mean (S.D)	24.4 (2.6)	24.3 (2.1)
Range	21.5–27.8	21–26.4

Five non-asthmatic females with a mean BMI of 24.3 ± 2.1 were recruited to the study as healthy controls. The control subjects had never smoked and had a mean age of 39.4 ± 10.3 years (Table 2). Two of the controls, Control_2 and Control_3, reported self-diagnosed dermatitis, although neither had received diagnosis by a physician for this condition.

### Inflammatory proteins

To determine the immune status of the asthmatic patients at the time of sample collection, qualitative ELISA was performed on the blood samples in order to profile the inflammatory state of the asthmatic and control, and inflammatory proteins under investigation included interleukin (IL)-4, IL-5, IL-10, IL-13, IL-17A, Eotaxin, Granulocyte-macrophage colony-stimulating factor (GM-CSF), Interferon gamma (IFNƴ  ), Monocyte chemo-attractant protein 1 (MCP-1), Thymus and activation regulated chemokine (TARC), and Tumour necrosis factor alpha (TNFA). Additionally, the concentration of the pro-inflammatory bacterial endotoxin protein was measured, and total IgE present in the blood was quantified to determine the atopic state of the asthmatic subjects.

**Figure 1:**
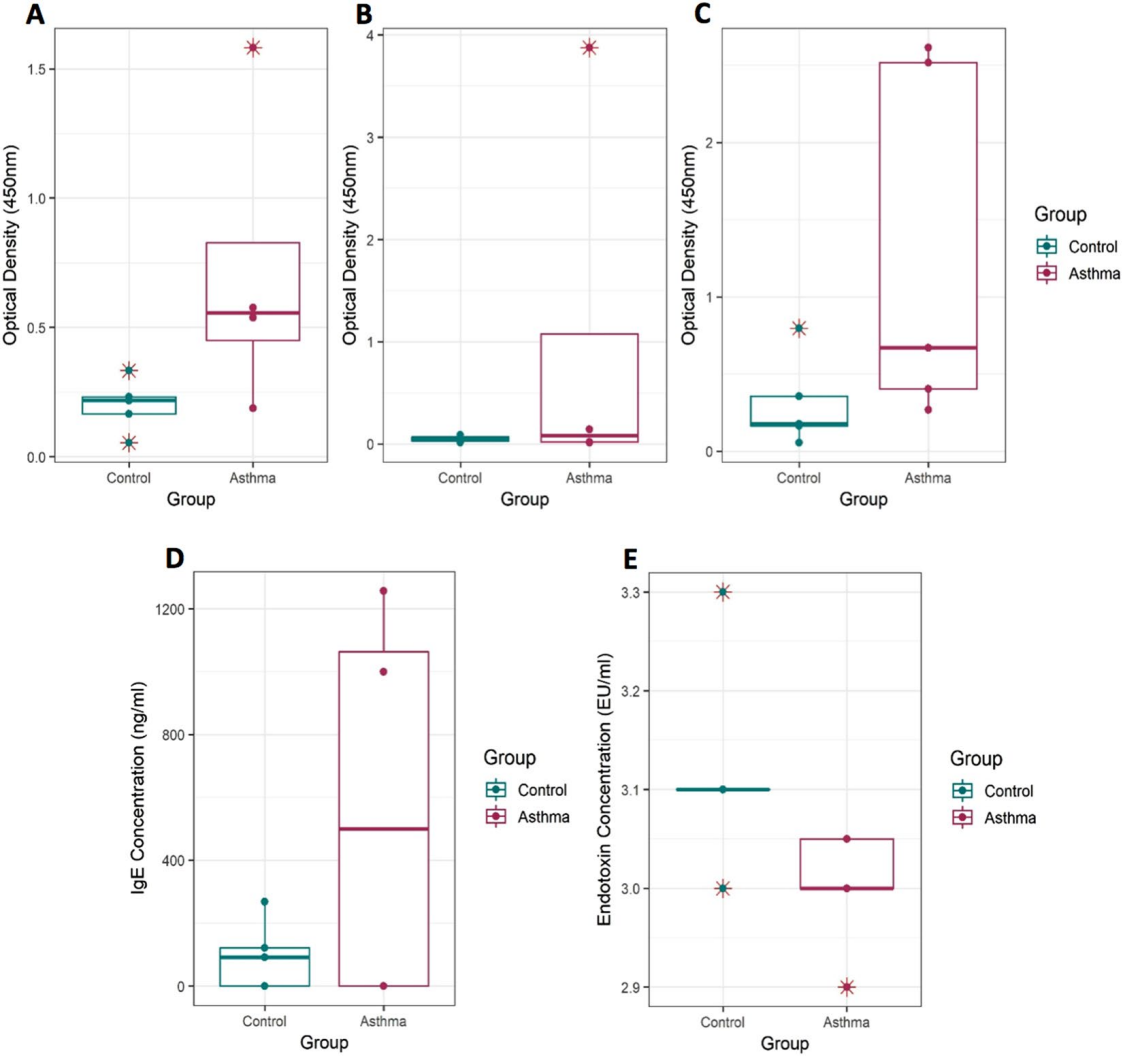
Analysis of circulatory inflammatory proteins present in blood samples from control subjects (n = 5) and asthma subjects (n = 5). **A** = levels of GM-CSF present in the blood of asthmatic subjects (n = 5) and control subjects (n = 5) using qualitative ELISA analysis, P value = 0.111 (Optical density, 450 nm; Wilcoxon rank sum test with continuity correction); **B** = levels of IFNƴ present in the blood of asthmatic subjects (n = 5) and control subjects (n = 5) using qualitative ELISA analysis, P value = 0.195 (Optical density, 450 nm; Wilcoxon rank sum test with continuity correction); **C** = levels of TARC in the blood using of asthmatic subjects (n = 5) and control subjects (n = 5) qualitative ELISA analysis, P value = 0.095 (Optical density, 450 nm; Wilcoxon rank sum test with continuity correction); **D** = Concentrations of total IgE protein present in the blood of asthmatic subjects (n = 4) and control subjects (n = 5) using quantitative ELISA analysis, P value = 1.0 (IgE concentration, ng/ml; Wilcoxon rank sum test with continuity correction); **E** = Concentrations of bacterial endotoxin present in the blood of asthmatic subjects (n = 5) and control subjects (n = 5) using Limulus Amebocyte Lysate (LAL) Chromogenic quantification. P value = 0.0650 (Endotoxin concentration, EU/ml; unpaired T test). EU/ml = endotoxin units per millilitre. Data points at 3.1 EU/ml for control = 3; Data points at 3.0 EU/ml for asthma = 2; Data points at 3.05 EU/ml for asthma = 2. * = Possible outliers, however due to the sample size, these were not excluded from our statistical analysis.

With regards to host-derived inflammatory proteins, 10 out of the 12 inflammatory proteins under investigation were detected in the blood samples (see Supplementary Materials, S3). Overall the asthmatic subjects were found to have elevated levels of inflammatory proteins compared to the controls, as determined by increased optical density (at 450 nm) for all inflammatory proteins examined. This was particularly apparent for the chemokines TARC (Fold change = 4.173; P value = 0.095), GM-CSF (Fold change = 3.607; P value = 0.111), and IFNƴ (Fold change = 20.871; P value = 0.195) (Fig. 1A–C). However, it should be noted that there were no statistically significant increases detected for any of the individual proteins. This was likely due to the asthmatic subjects having a greater level of diversity with regards to inflammatory protein levels compared to the control subjects (Fig. 1).

Total IgE was detected in 50% of the blood samples under investigation (three control subjects and two asthmatic subjects (Fig. 1D). For the purpose of statistical analysis, samples with undetectable levels of IgE were given an IgE concentration value of 0. Comparison between the concentrations of IgE detected in the asthmatic samples compared to the control samples revealed no significant differences. Endotoxin levels were found to be reduced in the asthmatic subjects (Fig. 1E; P value = 0.0650). Within the asthma cohort, subjects with additional atopic complications (i.e. allergic rhinitis, allergic dermatitis) displayed lower endotoxin concentrations compared to the asthmatic subjects that did not have additional atopic complications. Within the control cohort, subjects with previously reported atopic dermatitis displayed circulatory endotoxin concentrations similar (i.e. lower than those subjects reporting no atopic conditions) to those observed in the asthma cohort.

### mRNA sequencing and differential expression analysis

Approximately 20,000,000 messenger RNA (mRNA) read pairs were generated from each plasma sample (average 44,000,000 ± 3,100,000 reads), with no significant differences in read count identified between the two cohorts.

Expression of a total of 14, 226 genes was detected through assessment of the circulating transcriptome (i.e. those RNAs present in the plasma). Given the nature of our sample type, the extent of read mapping to key mRNAs was confirmed visually by appraising the resulting BAM file against hg19 using IGV (data not shown). Sample Asthma_2 failed to map satisfactorily to hg19 and was thus excluded due to concerns this would induce bias into our downstream analyses.

**Figure 2:**
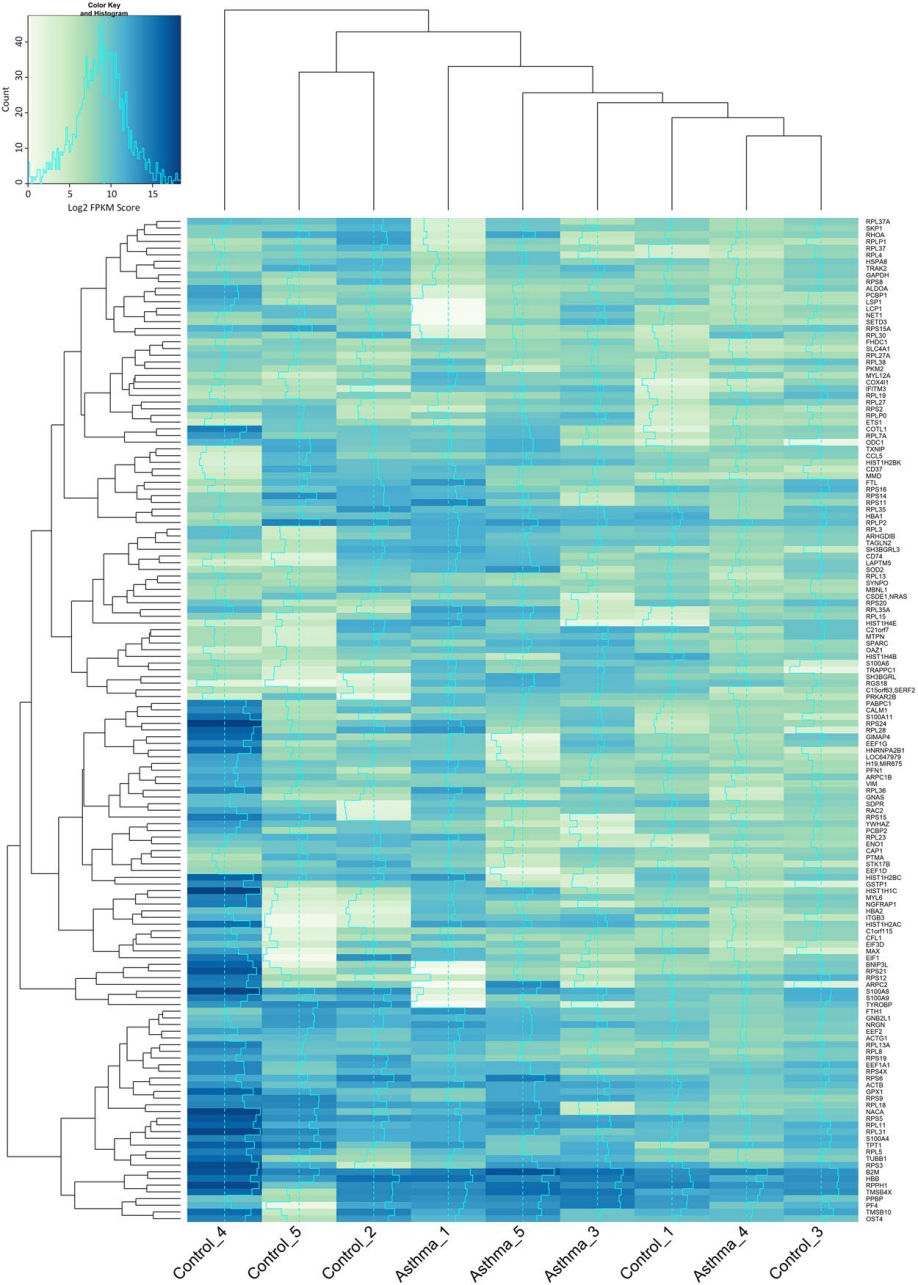
Heatmap showing highly expressed genes in control subjects (n = 5) and asthma subjects (n = 4). Gene expression is determined by quantification of circulatory mRNA present in the plasma samples and is expressed as log2 normalised Fragments Per Kilobase of transcript per Million mapped (FPKM) reads. Highly expressed genes, as determined by determined by a mean LOG2 FPKM score ≥7.0, were plotted, and Cluster analysis (Euclidean distance) informs the X and Y-axis dendrograms.

To determine whether the asthmatic subjects had a distinct gene expression profile compared to the control subjects, genes that displayed robust levels of expression (the top 150 most highly expressed genes as determined by a mean LOG2 FPKM score ≥7.0) were plotted as a heatmap and unsupervised clustering analysis performed using Euclidean distance (Fig. 2). Cluster analysis revealed that Control_4 had a relatively unique mRNA profile compared to the other subjects under investigation. For the remaining subjects, two clusters formed. Cluster one contained the control subjects Control_5 and Control_2, and Cluster two was made up of Asthma_1, Asthma_5, Asthma_3, Control_1, Asthma_4, and Control_3. Of note was Asthma_4, whose RNA profile more closely resembled the control subjects than the asthmatic subjects in Cluster two.

Statistical analysis, as detailed previously, revealed that 287 genes were differentially expressed in the asthmatic subjects (as defined by a Q ≤0.05 and a Log2 Fold Change >0.6). Within the asthmatic cohort, 90 of the differentially expressed genes showed significantly increased expression, and 197 genes displayed significantly decreased expression. Genes that displayed the highest degree of differential expression within the asthmatic subjects are listed in Table 3. A full list of differentially expressed genes can be viewed in the supplementary materials (Supplementary Materials, S4).

**Table 3:** The most differentially expressed genes in the asthmatic subjects (n = 4) compared to the control subjects (n = 5).

Gene	Control Mean	Asthma Mean	Fold Change (log2)	Q Value
**Downregulated Genes**				
DOHH	972.908	0	Control Only	0.002975
PTRH2	87.7907	0	Control Only	0.002975
C15orf41	79.1979	0	Control Only	0.002975
HIST1H3I	30.2331	0	Control Only	0.002975
HOXC10	26.4924	0	Control Only	0.002975
TSPYL5	18.9517	0	Control Only	0.002975
NFXL1	17.8423	0	Control Only	0.002975
RAB3IL1	15.1233	0	Control Only	0.002975
LINC00085	15.0233	0	Control Only	0.002975
ARV1	14.0641	0	Control Only	0.002975
**Upregulated Genes**				
HIST1H3C	0	90.5782	Asthma Only	0.002975
HDAC9	0.731644	52.1632	6.15575	0.005217
PRAM1	0	3.05743	Asthma Only	0.005217
PML	0.948462	178.238	7.554	0.007164
RAB6B	0	8.90346	Asthma Only	0.007164
NRP1	0.92425	18.8945	4.35354	0.010799
CD93	0	14.3366	Asthma Only	0.010799
GPR56	1.86976	98.5377	5.71975	0.012559
MR1	1.07632	17.8916	4.0551	0.017952
TOP1MT	0.344555	59.0342	7.42067	0.017952

Where genes are expressed in a condition-specific manner, Log2 fold change is replaced with “Control Only” or “Asthma Only” as appropriate. Quantity of the gene is shown as Fragments Per Kilobase of transcript per Million mapped (FPKM) reads.

To determine whether differential gene expression could be linked to asthma pathology, we compared the differentially expressed genes identified to a recently released database of genes associated with asthma pathology - AllerGAtlas, 2018^50^. Of the 287 genes identified as being significantly differentially expressed in the asthmatic subjects, 8 genes were identified in the asthma gene database. These genes included complement regulatory protein 46 (CD46), interleukin 7 receptor (IL7R), galactin 3(LGALS3), myeloperoxidase (MPO), neurotensin (NTS), phosphodiesterase 4 A (PDE4A), toll-like receptor (TLR) 1, and vitamin D receptor (VDR). Four of the genes were upregulated in the asthmatic subjects (VDR, NTS, TLR1, and MPO) and four were downregulated in the asthmatic subjects (LGAL3, CD46, IL7R, and PDE4A) (Table 4). Moreover, gene expression was predominately condition specific. Of the upregulated genes, NTS, TLR1, and MPO mRNA was only detectable in the asthma samples, whilst of the downregulated genes, IL7R and PDE4R mRNA were only observed in the control samples (see Supplementary Materials, S4).

**Table 4:** Genes with significant differential expression in the asthmatic subjects compared to control subjects that are associated with asthma pathology.

Gene	Expression in Asthma	Function	Reference
CD46	Downregulated	Differentiation of IL-10 producing regulatory T cell type 1 cells Differentiation of Th1 cells Inhibition of HDM allergenic activity	^51, 52^ ^53, 54^ ^55^
IL7R	Downregulated	Marker for Treg activation T cell development Eosinophil survival	^56^ ^57^
LGALS3	Downregulated	Inhibition of IL-5 expression Inhibition of eosinophil and T cell infiltration Negative regulation of Th17 polarization	^58^ ^58^ ^59^
MPO	Upregulated	Initiation of lipid peroxidation	^60^
NTS	Upregulated	Mast cell degranulation	^61, 62^
PDE4A	Downregulated	Production of CD4 + T cell cytokines (IL-2, IL-4, IL-5, IFNƴ ) Production of TNFα Production of leukotriene B4 Production of eotaxin Airway goblet cell hyperplasia	^63– 65^ ^63^ ^63^ ^65^ ^65^
TLR1	Upregulated	Antimicrobial activity	^66– 68^
VDR	Upregulated	Development of airway inflammation and hyper-responsiveness Eosinophilia Inhibits IgE production	^69^ ^69^ ^69, 70^

Differential gene expression was determined using the Tuxedo protocol (Galaxy software) on log2 normalised mRNA Fragments Per Kilobase of transcript per Million mapped (FPKM) reads sequenced from plasma samples from asthma (n = 4) and control subjects (n = 5). Gene function with regards to asthma pathology was determined using the database AllerGAtlas database, 2018^50^ and a general literature search using the relevant search engines employed to provide additional context.

### miRNA quantification

**Figure 3:**
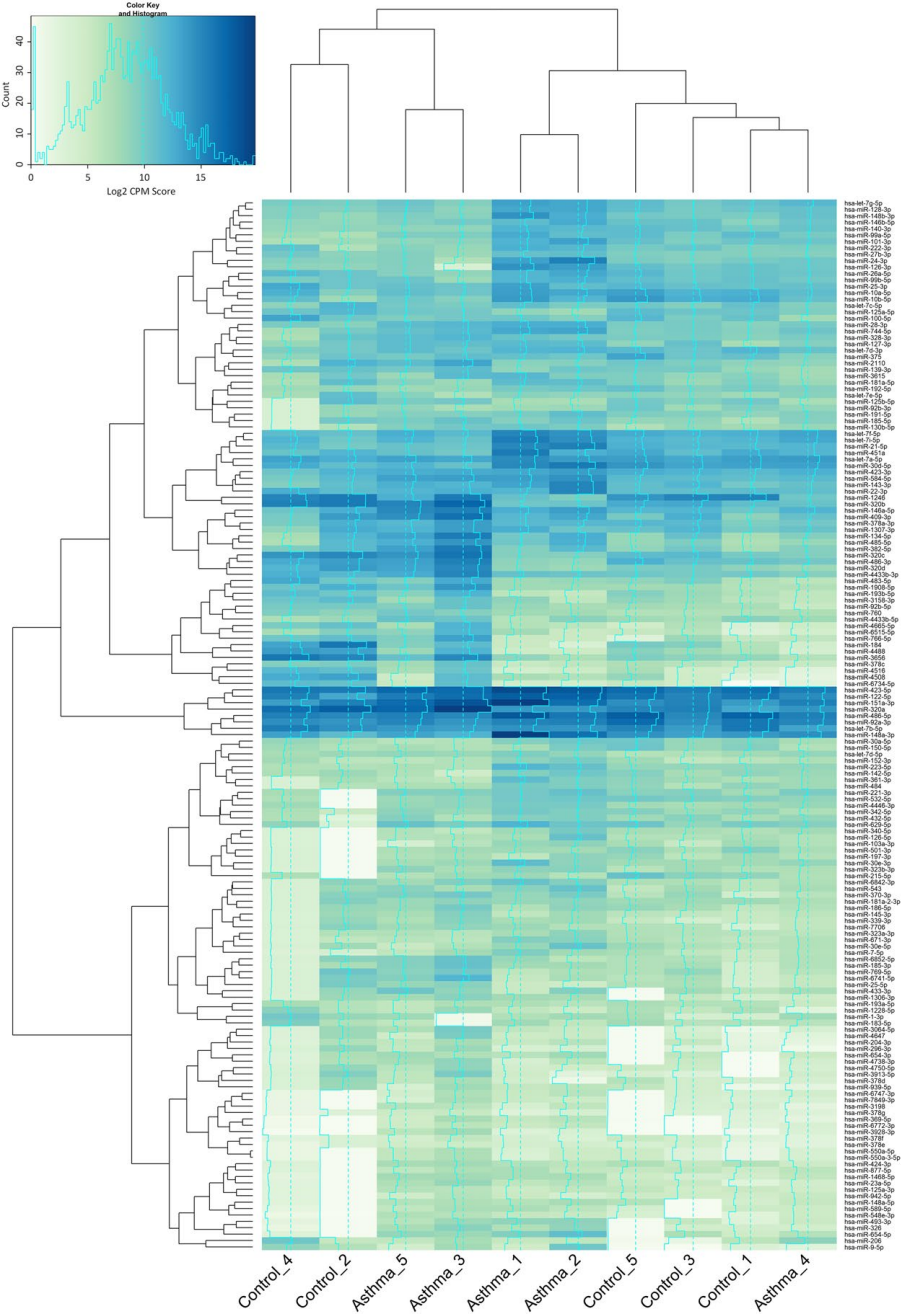
A Heatmap showing expression levels of circulatory miRNA in control subjects (n = 5) and asthmatic subjects (n = 5). miRNA expression is determined by quantification of circulatory miRNA detected in the plasma samples and is expressed as log2 normalised Counts per Million mapped (CPM) reads. Cluster analysis (Euclidean distance) informs the X and Y-axis dendrograms.

Approximately 10,000,000 microRNA (miRNA) reads were generated from each plasma sample (range = 10,276,765–16,812,591, mean = 12,030,581 ± 1,911,104), and there were no significant differences in read count identified between the control and asthma samples. Using miRanalyzer^44^ and edgeR^45^, we identified 166 known miRNAs present in the plasma samples (Fig. 3), which is consistent with previously reported studies^71–75^.

To determine whether the asthmatic state was associated with a distinct circulating miRNA profile, miRNAs with robust expression (as defined by a mean counts per million >4.0) were plotted as a heatmap, and unsupervised clustering performed using Euclidean distance (Fig. 3). Cluster analysis revealed the presence of two distinct clusters. Cluster one was composed of Control_4, Control_2, Asthma_5, and Asthma_3; and Cluster two was made up of Asthma_1, Asthma_2, Control_5, Control_3, Control_1, and Asthma_4. Within each cluster, two sub-clusters formed, and each sub-cluster was comprised of either control subjects or asthma subjects. Of interest was the observation that the asthmatic subjects with no additional atopic complications (Asthma_3 and Asthma_5) clustered together, and the asthmatics with additional atopic complications (Asthma_1 and Asthma_2) clustered together.

Statistical analysis revealed that 13 miRNAs were significantly increased (defined as FDR P value ≤ 0.05 and a fold change ≥2.0) in the asthmatic subjects compared to the control subjects (Supplementary Materials S5). Indeed, asthma was associated with increased circulating levels of miRNA-3928-3p, 6772-3p, 369-5p, 326, 151a-3p, 24-3p, 548e-3p, 1468-5p, 493-3p, 148a-3p, 654-5p, 382-5p, and 744-5p, and unsupervised hierarchical clustering (as above) based upon these 13 individual miRNA revealed two distinct clusters containing all control and all asthma subjects respectively.

**Figure 4:**
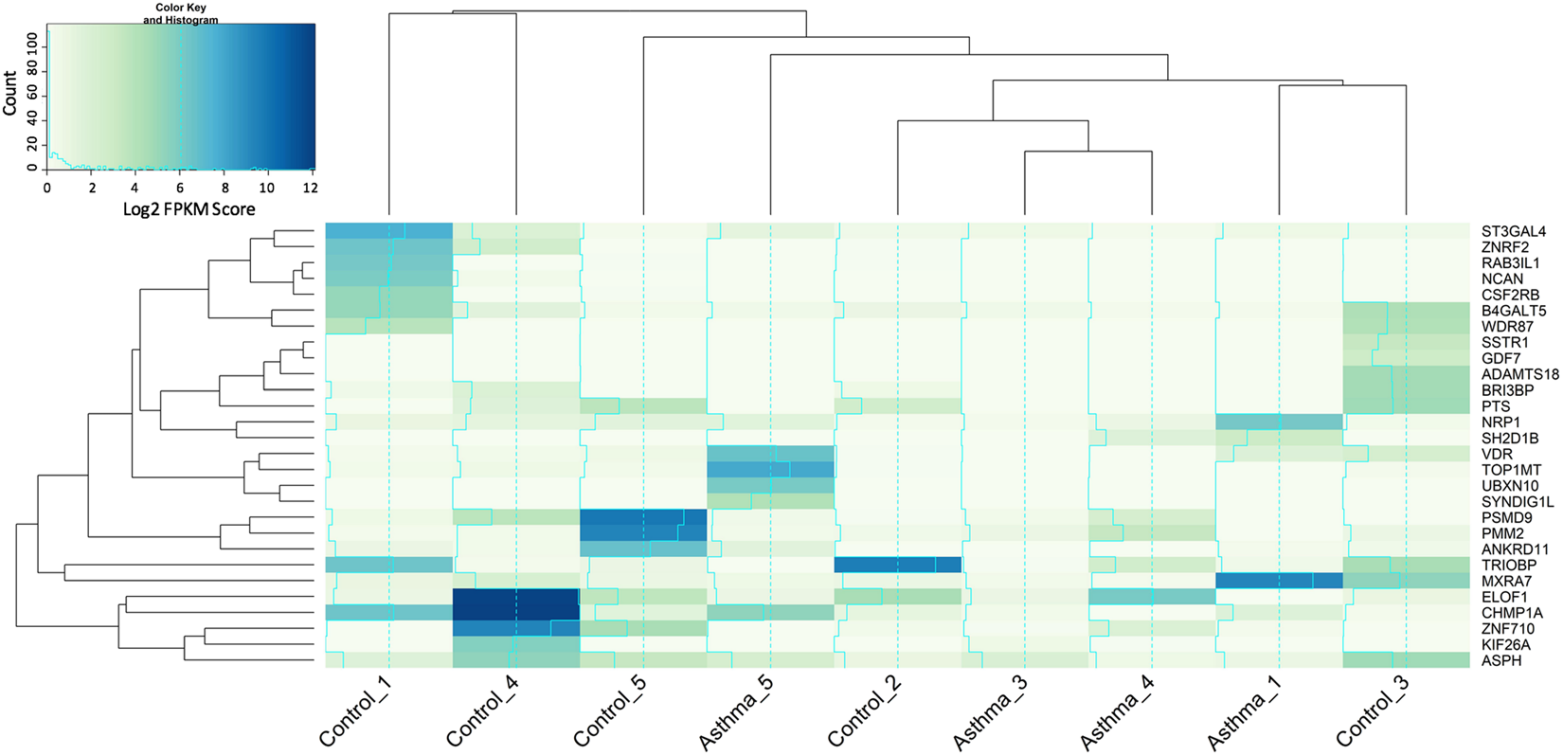
Genes targeted by the differentially expressed miRNAs that displayed significant differential expression in asthmatic subjects (n = 5) compared to control subjects (n = 5). mRNA expression was determined by quantification of circulatory mRNA detected in the plasma samples and is expressed as log2 normalised Fragments Per Kilobase of transcript per Million mapped (FPKM) reads. Significant differential expression was defined as having a log fold change greater than 2.0 and a false rate of discovery (FDR) adjusted P value ≤ 0.05. Identification of the gene as a target of differentially expressed miRNA was determined using TargetScan v6.2. Cluster analysis (Euclidean distance) informs the X and Y-axis dendrograms.

MicroRNAs are known regulators of gene expression, typically resulting in gene silencing. Using *in silico* predicted targets derived from TargetScan v6.2, a total of 1,831 genes were identified as being predicted targets of the differentially expressed (increased) miRNAs. Transcription of 1,324 of these target genes was detected in our plasma samples, of which 28 genes displayed significant differential expression in the asthmatic subjects (Fig. 4). The majority of these genes were significantly downregulated in the asthmatic subjects (21/28 genes), as expected.

Analysis of biological pathways likely impacted by the deregulated miRNAs identified 50 pathways likely to be altered in the asthmatic state. The top 15 biological pathways identified are shown in Table 5.

**Table 5:** Top 15 Biological pathways regulated by miRNAs that were differentially expressed in the asthmatic subjects (n = 5) compared to the control subjects (n = 5).

GO Category	P Value	# Genes	# miRNAs
organelle (GO:0043226)	1.18E-33	936	13
ion binding (GO:0043167)	2.90E-23	599	13
cellular nitrogen compound metabolic process (GO:0034641)	3.20E-15	445	13
biosynthetic process (GO:0009058)	3.13E-11	381	13
small molecule metabolic process (GO:0044281)	1.84E-07	226	12
neurotrophin TRK receptor signalling pathway (GO:0048011)	4.10E-07	35	8
protein binding transcription factor activity (GO:0000988)	1.41E-06	62	11
molecular_function (GO:0003674)	1.52E-06	1554	13
cytoskeletal protein binding (GO:0008092)	2.38E-06	92	12
blood coagulation (GO:0007596)	2.74E-06	55	10
gene expression (GO:0010467)	3.41E-06	61	10
cellular protein modification process (GO:0006464)	1.19E-05	214	13
synaptic transmission (GO:0007268)	4.93E-05	53	10
Fc-epsilon receptor signalling pathway (GO:0038095)	5.15E-05	22	8
cellular_component (GO:0005575)	5.40E-05	1562	13

### Functional analysis

Causal inference analysis using Ingenuity Pathway analysis (IPA) software was performed to identify the likely upstream regulators responsible for the changes in mRNA and miRNA expression noted in the asthmatic subjects.

In total, 246 upstream gene regulators had a P value of overlap ≤ 0.05; indicating that they have altered functional activity in the asthmatic subjects on the basis of differential mRNA and miRNA expression. Of these regulators, seven had Z scores greater than 2.0, thus enabling their activity to be predicted. Two upstream regulators were predicted to have significantly increased activity in the asthmatic subjects (P value of overlap ≤ 0.05; Z score ≥ 2.0), and five were predicted to have significantly decreased activity asthmatic subjects (P value of overlap ≤ 0.05; Z score ≤ −2.0) in the (Table 6).

**Table 6:** Upstream gene regulators with predicted significantly altered activity in the asthmatic subjects (n = 4) compared to the control subjects (n = 5).

Upstream Regulator	Molecule type	Activity state	Z score	P value of overlap	# Target molecules activated	# Target molecules inhibited	# Target molecules affected
Sirolimus	Chemical drug	Activated	2.75	0.0107	12	1	0
GFI1	Transcription regulator	Activated	2.00	0.0077	4	0	1
EIF4E	Transcription regulator	Inhibited	−2.00	0.0074	0	4	2
Mycophenolic acid	Chemical drug	Inhibited	−2.00	0.0211	0	4	0
Streptozocin	Chemical drug	Inhibited	−2.16	0.0492	0	5	1
SOX4	Transcription regulator	Inhibited	−2.24	0.0770	0	5	0
SYVN1	Transporter	Inhibited	−2.45	0.0069	0	6	0

Upstream regulators predicted to have significantly altered activity were defined as having a P value of overlap ≤ 0.05 and a Z score greater than 2.0. Activated upstream regulators are defined as having a Z score ≥ 2.0, and inhibited upstream regulators are defined as having a Z score ≤ −2.0. Target molecules activated = genes present in the RNA dataset that are activated by the upstream regulator; target molecules inhibited = genes present in the RNA dataset that are inhibited by the upstream regulator; target molecules affected = genes present in the RNA dataset whose activity is known to be altered by the upstream regulator but there is insufficient evidence to prove this is activation or inhibition.

### Downstream activity

Causal inference analysis using IPA was also used to predict the downstream consequences of the observed differential mRNA and miRNA expression within the asthmatic subjects. The downstream effects of the differential expression were primarily assessed by examination of the predicted canonical pathways and bio-functions impacted. Fourteen canonical pathways were found to have significantly altered biological activity (P ≤ 0.05) within the asthmatic subjects (Table 7). A number of canonical pathways predicted to be significantly altered in the asthmatic subjects influence immune activity, including T cell and B cell activity, phagosome maturation, signalling in rheumatoid arthritis, B cell development, and Nur77 signalling in T lymphocytes.

**Table 7:** Canonical signalling pathways predicted to have significantly altered activity in the asthmatic subjects (n = 4) compared to the control subjects (n = 5).

Canonical Pathway	P Value	Molecules with increased gene expression	Molecules with decreased gene expression
Altered T Cell and B Cell Signalling in Rheumatoid Arthritis	0.0053	SLAMF1,TLR1,HLA-DQA1,TNFRSF13C	HLA-DRB5
B Cell Development	0.0092	HLA-DQA1	IL7R, HLA-DRB5
Antigen Presentation Pathway	0.0116	HLA-DQA1, MR1	HLA-DRB5
Melatonin Degradation III	0.0124	MPO	—
TNFR1 Signalling	0.0241	—	TRADD, IKBKB, PAK4
Acute Myeloid Leukemia Signalling	0.0287	PML	CSF2RB, CEBPA, IDH3B
Tetrahydrobiopterin Biosynthesis I	0.0368	—	PTS
Hypusine Biosynthesis	0.0368	—	DOHH
Tetrahydrobiopterin Biosynthesis II	0.0368	—	PTS
Nur77 Signalling in T Lymphocytes	0.0369	HDAC9, HLA-DQA1	HLA-DRB5
Phagosome Maturation	0.0375	MPO, GOSR2	CTSL, CTSG, HLA-DRB5
Catecholamine Biosynthesis	0.0487	—	PNMT
Mitotic Roles of Polo-Like Kinase	0.0488	STAG2	ANAPC4, PPP2R5C
Type I Diabetes Mellitus Signalling	0.0496	HLA-DQA1	TRADD, IKBKB, HLA-DRB5

Causal interference using Ingenuity Pathway Analysis (IPA) software was used to predict downstream canonical signalling pathways likely to be affected by changes in gene expression and regulation in the asthmatic subjects. Molecules with increased gene expression are genes that had significantly increased numbers of mRNA reads in the asthma plasma samples, and molecules with decreased gene expression are genes that had significantly decreased numbers of mRNA reads in the asthma plasma samples. Canonical pathways that are defined as being significantly altered in the asthma subjects have a P value ≤ 0.05.

### Bio-function analysis

With regards to biological functions likely to be impacted by changes in the observed mRNA and miRNA expression patterns, a number of key immunological pathways were predicted to have altered activity within the asthmatic cohort (Table 8).

**Table 8:** Biological functions predicted to have significantly altered activity in the asthmatic subjects (n = 4) compared to the control subjects (n = 5).

Biological Functions	P Value	Activation State	Z score
Binding of endothelial cells	9.75E-03	Decreased	−2.123
Binding of leukocytes	1.73E-03	Decreased	−2.062
Cell transformation	1.32E-03	Decreased	−3.228
Differentiation of fibroblast cell lines	4.44E-03	Decreased	−2.184
Immune response of leukocytes	6.79E-04	Decreased	−2.031
Interaction of endothelial cells	3.55E-03	Decreased	−2.346
Killing of natural killer cells	5.44E-03	Decreased	−2.63
Proliferation of hepatocytes	6.53E-03	Increased	2.177
Tumorigenesis of tissue	4.94E-04	Increased	2.215
Viral infection	1.34E-02	Decreased	−2.099

Causal inference using Ingenuity Pathway Analysis (IPA) software was used to predict biological functions likely to have altered activity in the asthmatic subjects. This was determined through analysis of genes and miRNA that had altered expression in the asthmatic subjects, to predict which biological functions would likely be altered. Biological functions predicted to be significantly altered in the asthmatic subjects were defined as having a P value ≤ 0.05 and a Z score greater than 2.0. Biological functions with predicted increased activity were defined as having a Z score ≥2.0, and biological functions with predicted decreased activity were defined as having a Z score ≤−2.0.

### Characterisation of the blood microbiome

**Figure 5:**
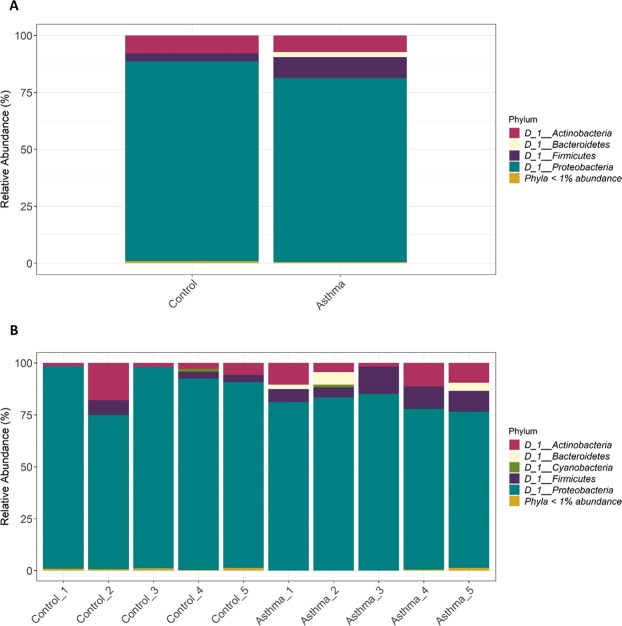
Microbial profile of the blood microbiome at the phylum level in asthmatic subjects (n = 5) and control subjects (n = 5). Composition of the blood microbiome was determined through sequencing of the bacterial V4 region of the 16S rRNA gene from bacterial DNA isolated from plasma samples from control subjects (n = 5) and asthmatic subjects (n = 5). The generated bacterial sequences were clustered (99% identity) in Operational Taxonomic Units (OTUs) to the Silva database and then assigned to bacterial taxonomic classes. **A** = microbial profile of the asthmatic subjects (n = 5) compared to the control subjects (n = 5). **B** = Microbial profiles of the individual plasma samples (n = 10). Note, this figure has been published previously as part of a separate study comparing molecular methods of blood microbiome characterisation. Further details can be found here^47^.

**Figure 6:**
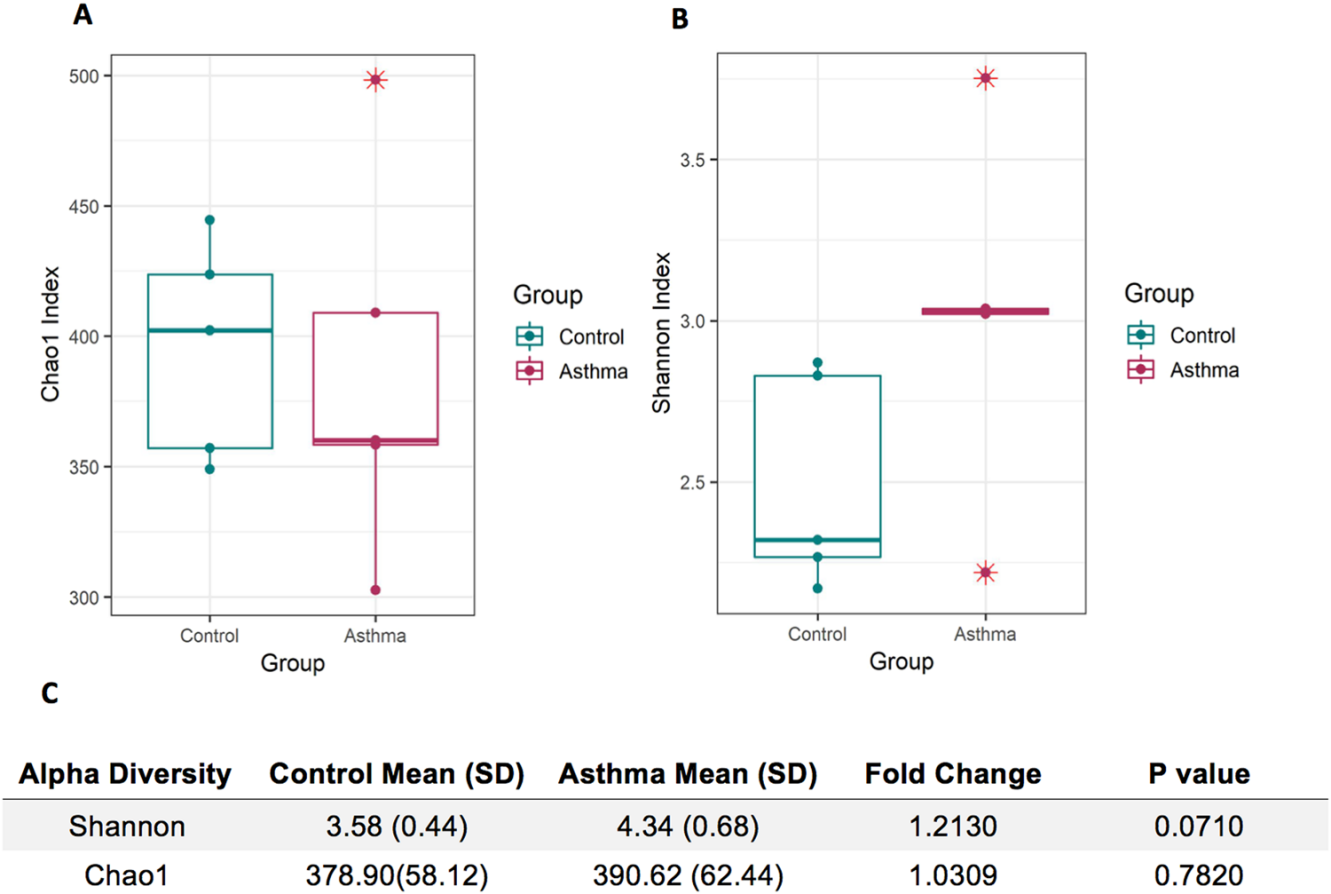
Comparison of alpha diversity present in the asthma blood microbiome compared to the control blood microbiome. Alpha diversity was measured using rarefied OTU tables generated from 16S rRNA sequencing data from plasma samples collected from asthma subjects (n = 5) and control subjects (n = 5). Shannon diversity index scores were generated from OTU tables in order to measure the richness and evenness of bacterial taxa present in the plasma samples. Chao1 index scores were measured to determine the predicted number of bacterial taxa present in the plasma samples by extrapolating out the number of rare organisms that may not have been detected due to under-sampling. **A** = Comparison of Shannon index scores generated from asthma plasma samples (n = 5) and control plasma samples (n = 5), **B** = Chao1 index scores generated from asthma plasma samples (n = 5) and control plasma samples (n = 5). **C** = Statistical analysis of alpha diversity detected in the asthma plasma samples (n = 5) and control plasma samples (n = 5). SD = standard deviation. * = Outlier.

Our previous characterisation of bacterial nucleic acid present in the plasma samples studied herein^47^ found that the majority was from the *Proteobacteria* phylum (Total relative abundance = 83.9%; Control mean = 90.0%; Asthma mean = 80.3%), the *Actinobacteria* phylum (Total relative abundance = 7.5%, Control mean = 6.0%, Asthma mean = 7.5%), and the *Firmicutes* phylum (Total relative abundance = 6.6%, Control mean = 3.0%, Asthma mean = 9.0%) (Fig. 5, reproduced from^47^). Please refer to^47^ for a detailed appraisal of our experimental controls. At the genus level, 81 bacterial genera were detected in the asthma plasma samples compared to 49 bacterial genera detected in the control plasma samples. Alpha and beta diversity of the bacterial populations present in the asthma and control groups was therefore assessed to determine whether there was significantly altered bacterial diversity within the blood microbiome of the asthmatic subjects. Asthmatic subjects scored higher Chao1 and Shannon index scores than the control subjects (Fig. 6; Supplementary Material S6 for individual sample scores). This was particularly apparent for the Shannon diversity scores (P value = 0.0710) (Fig. 6A,C). Of note was the observation that Asthma_3 had a Shannon diversity score more similar to the controls than to the other asthmatic subjects (Supplementary Material S6).

Statistical analysis of community composition revealed that asthma was associated with a significant increase in *Firmicutes* (P value = 0.0148), and a concomitant decrease in *Proteobacteria* (P value = 0.0702) (Fig. 5, reproduced from^47^). To a lesser extent, members of the *Bacteroidetes* phylum were also detected in the blood samples, with increased levels of *Bacteroidetes* observed in the asthmatic subjects (Control mean relative abundance = 0.26%, range = 0.0–2.7%; Asthma mean relative abundance = 2.40%, range = 0–6.0%), although this increase was not statistically significant.

### Lefse analysis

**Figure 7:**
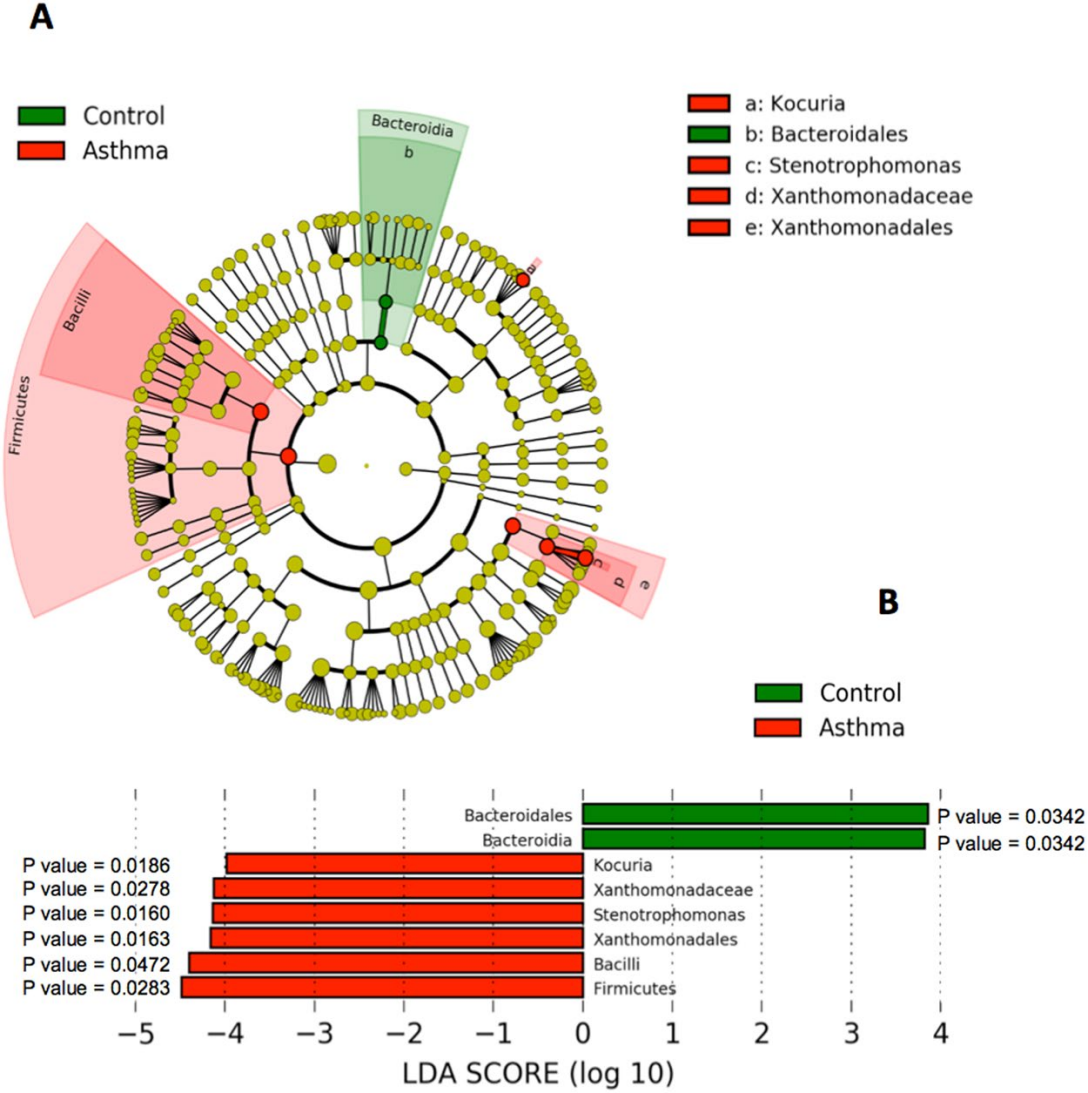
Comparison of the healthy blood microbiome (n = 5) and the asthmatic blood microbiome (n = 5) using LefSe. Linear discriminant analysis effect size (Lefse) analysis was performed on the bacterial taxa relative abundance values to determine the presence of bacterial taxa with statistically significant changes in abundance in the asthma blood microbiome compared to the control blood microbiome. (**A**) Taxonomic cladogram showing control enriched taxa (Green) and asthma enriched taxa (Red). (**B**) Effect size of the differential taxa. The control enriched taxa are indicated with a positive LDA score, and the asthma enriched taxa are indicated with a negative LDA score. The level of significance is indicated by the P value shown for each taxa.

The above conventional statistical approach informed by relative abundance data collected at the phylum level reported significant differences in the blood microbiome between control and asthma subjects. To explore this finding further, linear discriminant analysis effect size (LefSe) was applied to 16S rRNA relative abundance data at all taxonomic levels to determine the bacterial taxa most likely to explain the differences between the control and asthma blood microbiomes. LefSe was also used to determine the biological consistency and effect relevance of the observed differences in relative abundance. LefSe identified 8 bacterial taxa that showed statistically significant and biologically consistent differences in the asthmatic subjects compared to control (Fig. 7). The taxa *Bacteroidales* and *Bacteroidia* were associated with the control state. Conversely, the taxa *Kocuria*, *Xanthomonadaceae*, *Stenotrophomonas*, *Bacilli* and *Firmicutes* were associated with the asthmatic state. These findings are consistent with our previous analysis of the bacterial populations using standard statistical tests (complete data not shown).

## Discussion

This study aimed to characterise a small yet specific population of female asthma patients who had developed atopic asthma during childhood. A range of molecular techniques were applied to characterise gene expression and regulation, inflammatory protein levels, and nucleic acid evidence of bacteria present in the blood. This was carried out in an effort to increase our understanding of this particular asthma phenotype, to begin to explore the molecular mechanisms responsible, and to identify any candidate biomarkers for future study.

At the protein level, asthma was associated with increased inflammatory protein levels detected in the blood. This was particularly apparent for GM-CSF, IFNƴ, and TARC. The range of inflammatory protein levels detected within the asthmatic cohort was noticeably higher than that observed in the control cohort. This was explained by the presence of two distinct clusters in the asthmatic cohort; cluster one was composed of subjects Asthma_2 and Asthma_4, and was characterised by high inflammatory protein levels; and cluster two was composed of Asthma_1, Asthma_3, and Asthma_5, and was characterised by lower levels of inflammatory proteins that were similar to those observed for the control subjects.

Of particular interested was IL-17A; this protein was found to be present at higher levels in the asthmatic subjects who suffered additional atopic complications (Asthma_1, Asthma_2, and Asthma_4) and the two control subjects who had self-reported atopic dermatitis (Control_2 and Control_3) (see Supplementary Material S2 and S3). This finding is supported by previous studies that have reported increased levels of IL-17A associated with asthma severity^76–80^, the Th2 immune response^81,82^, and atopic dermatitis^81–83^. Moreover, increased levels of IL-17A in asthmatic subjects has been associated with treatment response^79^. The association of IL-17A with the various asthma phenotypes, therefore, warrants further investigation.

Measurement of IgE concentration revealed that IgE was only detectable in half of the blood samples investigated (3 control subjects, and 2 asthmatic subjects). The low detection rate of IgE was not unexpected given its short half-life (approximately two days) and known low concentration levels within the blood^84^. There was no significant increase in IgE concentration in the asthmatic subjects however this is likely due to the small number of samples with detectable IgE. However, it was observed that IgE was detected in asthma subjects belonging to the proposed cluster one (Asthma_2 and Asthma_4) i.e. those with additional complications from the eosinophilic triad. This further supports the theory of asthmatic subjects forming sub-phenotypes.

In contrast to IgE, endotoxin concentration was decreased in the asthmatic subjects (P value = 0.0650), and there appeared to be an inverse correlation between circulatory endotoxin levels and the reporting of additional atopic complications with those patients with more conditions from the eosinophilic triad, having lower levels than those patients with asthma alone and the control patients. Exposure to endotoxin during early childhood has been previously reported to be protective of the development of childhood asthma^85–89^. This association is thought to specifically affect the sensitisation stage of atopic asthma. In rats, for example, Tulic *et al*. demonstrated that the protective effects of endotoxin only occurred when rats were exposed to endotoxin prior to ovalbumin sensitisation^90^. As this study was conducted on a cohort of adult asthmatics, our data suggest that the association between reduced endotoxin concentration and asthma may persist into adulthood and the chronic, life-long form of the disease. Reduced endotoxin levels may also be maintained due to changes in immune activity as a consequence of the disease. Increased levels of IL-17A have been positively associated with expression of anti-microbial peptides^91,92^. It is therefore interesting to speculate that increased IL-17A levels, as a result of asthma, may alter the composition of the asthmatic microbiome, resulting in reduced *Proteobacteria* colonisation, and subsequent reduction of endotoxin levels (see later), a protein typically produced by *Proteobacteria*. This interpretation is further supported by our RNA analysis, which revealed the upregulated expression of MPO and TLR in our asthmatic subjects. Both genes encode proteins involved in antimicrobial activity (Myeloperoxidase and Toll-like receptor 1, respectively), and thus upregulation of these genes has the potential to influence microbiome composition.

Analysis of the diversity of RNA expression within the blood revealed that our asthmatic donors had more similar RNA profiles to one another than they did to the control subjects; this was particularly apparent in the miRNA analysis. When combined with our differential expression analyses, we identified specific mRNA and miRNA populations within the blood that were distinct between the asthma and control cohorts. Asthma severity was found to influence gene expression, whilst miRNA expression appeared to be influenced by the presence of additional atopic complications.

Furthermore, it was intriguing to note that Asthma_4 displayed similar mRNA and miRNA profiles to the control subjects. This asthmatic subject was the youngest member of the asthma cohort, with an age of 19 years, and the subject had been suffering from asthma for just 14 years compared to the mean length of 38 years that our other subjects had been living with the disease. It is tempting to speculate that asthmatic mRNA profiles become more divergent from control profiles as the disease progresses over time, however our sample size restricts further analysis of this. Moreover, at the time of sample collection Sample_4 was suffering from the highest number of atopic diseases from the eosinophilic triad (atopic asthma, atopic dermatitis, and allergic rhinitis). It is possible that the presence of atopic dermatitis and allergic rhinitis would have additive effects on the protein, RNA, and microbial profile, which could explain why this individual differed to the other asthmatic subjects. That said, it is surprising that the individual displayed more similarity to the control subject, and thus further study is warranted to determine whether age, the presence of additional atopic complications, or indeed variations in the medication taken were responsible for the variation observed. With regards the unmet need for asthma biomarkers, we identified various mRNAs in the circulation that were uniquely expressed in the asthmatic subjects, including HIST1H3C, PRAM1, RAB6B and CD93. Of these, elevated levels of soluble CD93 have been previously reported in the serum of asthmatics during acute asthma exacerbations^93^ and in the serum of steroid-naïve asthmatic patients^94^ and this mRNA would therefore warrant further investigation.

Investigation of differentially expressed mRNAs using the AllerGAtlas asthma gene database found that 8 genes had been previously found to influence asthma pathogenesis. These genes have been demonstrated to influence a number of key components of asthma pathology, including eosinophil and T cell migration, production of Th2 cytokines (IL-4, IL-5, and IL-13), mast cell degranulation, IgE production, and airway hyperresponsiveness. Moreover, several of the downregulated genes (CD46, IL7R), have been found to have roles in Treg differentiation and activation. These cells are important regulators of T cell, and thus downregulation of CD46 and IL7R suggests loss of control of T cell activity in the asthmatic subjects. It was further observed that a number of genes detected at altered levels in the asthmatic subjects have antimicrobial activity, thus suggesting that immune dysregulation was influencing how the asthmatic immune system responds to microbes.

Small RNA sequencing and analysis identified 13 miRNAs that were all increased in the asthmatic state. Of these, many have been previously associated asthma, elements of asthma pathology, or other eosinophilic conditions. MicroRNA 148 has been identified as a candidate biomarker of asthma and allergic rhinitis^74^ whilst miR-382 has been proposed as a biomarker of asthma and COPD^95^. MicroRNA 548 is deregulated in asthma bronchial epithelial cells^96^ and mir-744 has been found to be elevated in a murine model of chronic asthma^97^. MicroRNAs 151a and 24 have been implicated in the pathogenesis of atopic dermatitis^98^ and the regulation of allergic inflammation^99^ respectively. MicroRNA-326 has been reported to regulate the profibrotic functions of Transforming growth factor beta in pulmonary fibrosis^100^. These deregulated miRNAs were determined to regulate the expression of 1,831 genes. This finding was reflected in the mRNA data, whereby the asthmatic subjects displayed significant downregulation of genes compared to the control subjects. With regards to asthma pathology, changes in miRNA expression was predicted to significantly affect IgE signalling. IgE signalling is known to be crucial in allergen sensitisation and subsequent atopic disease, and thus it is predicted that miRNA profiles would have an important role the initial development of asthma and subsequent allergen-induced asthma exacerbations. It would, therefore, be interesting to determine if what we observed in the miRNA profiles in adult asthmatics also occurs in asthmatic children. Moreover, as there are distinct differences in asthma prevalence and severity between the two sexes, it would be beneficial to repeat our study using a male cohort to determine if similar miRNA profiles would be present in male asthmatics.

When functional analysis was performed to determine the combined effects of altered mRNA and miRNA expression, a number of immune functions were predicted to be significantly altered. With regards to upstream regulators predicted to have altered activity in the asthmatic subjects, the activated state of GFI1 was of interest. GFI1 is known to enhance Th2 expansion^101^, and thus predicted activation of this transcription regulator would suggest increased T cell activation and subsequent expansion of the Th2 cell populations within the asthmatic cohort. This notion is further supported by the prediction of significant inhibition of the upstream regulator SOX4 in the asthmatic cohort. This transcription factor has been observed to suppress Th2 differentiation^102^, and thus its inhibition would allow expansion of the Th2 populations within the asthmatic subjects. Moreover, the predicted activated state of GFI1 would also influence innate immune responses within the asthmatic cohort. The transcription factor has been found to have a role in the development and maintenance of type 2 innate lymphoid cells^103^; a cell population that has been found to be involved in allergic lung inflammation^104–106^. When the predicted downstream effects of altered gene and miRNA expression was analysed similar findings of predicted immune dysregulation were observed. A number of canonical pathways involved in T cell and B cell activity, including signalling in rheumatoid arthritis, B cell development, and Nur77 signalling, were predicted to be significantly affected by the differential expression of mRNA and miRNA in the asthmatic cohort. It is interesting to note that the canonical pathways involved in rheumatoid arthritis and Type 1 diabetes were identified, as both diseases have been found to display co-occurrence with asthma^107,108^. It is tempting to speculate that presence of similar/shared underlying immune pathologies in the three diseases.

Unsurprisingly, a number of biological functions were predicted to be altered as a consequence of changed upstream activity and altered canonical signalling. Leukocyte activity was identified as being decreased in the asthmatic cohort. However, at this level of analysis, the downstream effects on biological function of the different classes of leukocytes was not determined, and thus further study would be required to ascertain which leukocytes would likely have altered activity in the asthmatic subjects as a consequence of the differential mRNA and miRNA expression. Study of the specific leukocyte classes affected by asthma would be crucial, as inhibition of the Th1 or Treg lymphocytes would likely enhance asthma pathophysiology, whereas inhibition of the Th2 lymphocytes would likely alleviate asthma pathophysiology.

It was also of interest to observe the predicted decrease in killing of natural killer cells. This cell population has been previously identified as having a critical role in immune defence against viruses and bacteria^109–112^. In particular, viral infections have been long characterised to exacerbate asthma^113–116^, and asthmatics have been observed to be deficient in type I IFN production^117–119^, which likely influences natural killer cell activity. Moreover, in a murine model, natural killer cell activity was found to be decreased during a Th2 response^120^. This suggests that in asthmatic subjects, as a consequence of a Th2 biased immune system, there is reduced natural killer cell activity, resulting in the known associations with asthma and respiratory infections. Moreover, this may also partially explain the changes in the airway microbiome we see in asthmatic populations.

Our previously published microbial characterisation^47^ informed by 16S rRNA amplification and sequencing, was re-analysed herein and revealed increased levels of *Firmicutes* and decreased levels of *Proteobacteria* within the blood of our asthmatic donors. This finding was accompanied by increased bacterial diversity within the blood of asthmatic subjects, and the identification of several additional bacterial taxa displaying significantly altered levels dependent on disease state. The observed decrease in circulating *Proteobacteria* rRNA in the asthmatic state is thought to be indicative of reduced *Proteobacteria* carriage within the asthmatic subjects at a distant microbiome niche (e.g. the gut, airways and oral cavity). This may explain the decreased levels of endotoxin (protein) detected in our asthmatic subjects, given that endotoxin-producing gram-negative bacteria dominate this phylum. Previous studies have associated childhood asthma and reduced endotoxin exposure, and it is interesting to note that we detected this same phenomenon in our adult asthma cohort, many years following childhood. Furthermore, our asthma patients were found to have increased levels of *Bacteroidetes* rRNA, and this appeared to be dependent on medication status with those patients taking anti-inflammatory medications having lower levels of circulating *Bacteroidetes* 16S rRNA than those who were not. As blood circulates the body and functions as a medium that samples from virtually all body sites^121^, it was not possible to determine herein the microbial niche from which these signals originated. That said, we hypothesise that changes in the blood are reflective of dysbiosis at distant site(s) with well-characterised microbial communities (e.g. the gut, oral cavity and skin), and have significant biomarker potential. In support of this interpretation, studies investigating the asthmatic airway microbiome have observed reduced *Firmicutes* in the asthmatic airways compared to healthy control subjects^122,123^. It is therefore possible that the increased levels of *Firmicutes* RNA we observed in the blood is a consequence of increased translocation of these bacteria and/ or their DNA from the airways into the blood compared to control subjects.

In addition to changes in bacterial abundance, asthmatic subjects also displayed increased bacterial diversity in the blood compared to control subjects. Increased bacterial diversity has also been observed in the asthmatic airways compared to healthy controls^122,124^. This is likely a consequence of the immune dysregulation that typically occurs in the asthmatic lung and suggests that immune activity in the airways influences bacterial diversity in the airways. However, it is also possible that immune dysregulation in the airways and blood as a result of atopic asthma has an adverse effect on the immune system’s ability to detect and control colonisation of bacteria in these body habitats. This would explain the apparent bacterial expansion we detected in the blood.

This study provides a valuable insight into the systemic changes evident in the HDM-associated asthma, identifies a range of molecules that are present in the circulation in a condition-specific manner (with clear biomarker potential), and highlights a range of hypotheses for further study. Moreover, our data also provide an insight into the level of heterogeneity observed both within the control and asthma samples investigated and will be of use for informing sample size calculations for future studies.

### Research affiliations

The National Institute for Health Research Health Protection Research Unit (NIHR HPRU) in Health Impact of Environmental Hazards at King’s College London in partnership with Public Health England (PHE) in collaboration with Imperial College London.

### Preprint

A pre-print of this publication has been deposited in bioRxiv: BIORXIV/2018/446427.

## Supplementary information

Supplementary Data 1
