# Supplementary material for: Multi-Method Molecular Characterisation of Human Dust-Mite-associated Allergic Asthma
Source: Sci Rep. 2019 Jun 20;9:8912. doi: 10.1038/s41598-019-45257-1 (PMC6586825; doi:10.1038/s41598-019-45257-1)
Supplement: Supplementary file 1 — Supplementary Data 1 [file 41598_2019_45257_MOESM1_ESM.docx]

**Supplementary Materials**

Multi-Method Molecular Characterisation of Human Dust-Mite-associated Allergic Asthma

E. Whittle^1^, M.O. Leonard^2^, T.W. Gant^2^ and D.P Tonge^1^

^1^School of Life Sciences, Faculty of Natural Sciences, Keele University, ST5 5BG

^2^Centre for Radiation, Chemical and Environmental Hazards, Public Health

England, OX11 0RQ

**S1:** Oligonucleotide primer sequences used in the PCR of the bacterial V4 region of the 16S rRNA gene

| **Primer Name** | **Sequence (5’ – 3’)** | **Length** |
| --- | --- | --- |
| V4_F | GTGCCAGCMGCCGCGGTAA | 19 |
| V4_R | GGACTACHVGGGTWTCTAAT | 20 |
| V4_XT_F | TCGTCGGCAGCGTCAGATGTGTATAAGAGACAGGTGCCAGCMGCCGCGGTAA | 52 |
| V4_XT_R | GTCTCGTGGGCTCGGAGATGTGTATAAGAGACAGGGACTACHVGGGTWTCTAAT | 54 |
|  | | |

**S2:** Characterisation of the Allergic Asthma cohort (n = 5) at the time of sample collection

| **Characteristic** | **Asthma_1** | **Asthma_2** | **Asthma_3** | **Asthma_4** | **Asthma_5** |
| --- | --- | --- | --- | --- | --- |
| **Current age** | 52 | 36 | 42 | 19 | 49 |
| **Ethnicity** | Hispanic | Hispanic | Caucasian | Caucasian | Hispanic |
| **BMI** | 27.8 | 27.3 | 23.3 | 21.5 | 22.3 |
| **Age of diagnosis** | 7 | 4 | 12 | 5 | 3 |
| **Asthma Diagnosis** | Allergic Asthma | Allergic Asthma | Allergic Asthma | Allergic Asthma | Allergic Asthma |
| **Years with disease** | 45 | 32 | 30 | 14 | 46 |
| **ACQ Score** |  |  |  |  |  |
| Total | 12 | 11 | 11 | 10 | 10 |
| Mean | 2.0 | 1.8 | 1.8 | 1.7 | 1.7 |
| **Pulmonary test score** | 65 | 61 | 70 | 78 | 68 |
| **Allergy** | House dust mite | House dust mite | House dust mite | House dust mite | House dust mite |
| **Other allergic conditions** | Allergic Rhinitis, Nasal Polyps | Allergic Rhinitis, Polycystic Ovary | None | Allergic Rhinitis, Allergic Dermatitis | none |
| **Medications** | Dulera, Albuterol | Dulera, Albuterol | Symbicort, Zyrtec, Albuterol | Albuterol, Qvar | Albuterol |
| **Collection Time** | 9:50am | 9:20am | 12:10pm | 10:30am | 10:55am |

**S3: Analysis of circulatory inflammatory proteins present in blood samples from asthmatic subjects (n = 5) and control subjects (n = 5)**. Analysis performed using qualitative ELISA. BLD = below level of detection; ALD = above level of detection. IL = interleukin, GM-CSF = granulocyte-macrophage colony-stimulating factor, IFNƴ = interferon gamma, MCP-1 = monocyte chemoattractant protein-1, TARC = thymus- and activation-regulated chemokine/ CCL17, TNFA = tumor necrosis factor alpha

|  | **Protein level (Optical density at 450 nm)** | | | | | | | | | |
| --- | --- | --- | --- | --- | --- | --- | --- | --- | --- | --- |
|  | **IL-4** | **IL-5** | **IL-10** | **IL-17A** | **Eotaxin** | **GM-CSF** | **IFNy** | **MCP-1** | **TARC** | **TNFA** |
| Control_1 | 0.001 | BLD | 0.008 | 0.032 | 0.210 | 0.217 | BLD | BLD | 0.178 | 0.133 |
| Control_2 | 0.016 | BLD | 0.003 | 0.168 | 0.146 | 0.165 | 0.011 | BLD | 0.165 | 0.038 |
| Control_3 | 0.046 | 0.075 | 0.036 | 0.372 | 1.142 | 0.232 | 0.086 | 0.143 | 0.796 | 0.237 |
| Control_4 | 0.013 | 0.003 | 0.006 | 0.084 | 0.270 | 0.333 | BLD | BLD | 0.357 | 0.051 |
| Control_5 | 0.019 | 0.004 | 0.008 | 0.004 | 0.215 | 0.054 | BLD | BLD | 0.055 | 0.042 |
| Asthma_1 | 0.022 | 0.008 | 0.009 | 0.356 | 0.454 | 0.187 | BLD | BLD | 0.268 | 0.063 |
| Asthma_2 | 0.202 | 0.055 | 0.164 | ALD | 1.423 | ALD | 3.877 | 0.617 | 2.614 | 0.619 |
| Asthma_3 | 0.002 | BLD | 0.005 | 0.094 | 0.137 | 0.538 | 0.011 | BLD | 0.404 | 0.071 |
| Asthma_4 | 0.269 | 0.020 | 0.043 | 1.858 | 0.529 | 1.582 | 0.142 | 0.035 | 2.516 | 0.212 |
| Asthma_5 | 0.019 | 0.005 | 0.014 | 0.068 | 0.207 | 0.576 | 0.019 | BLD | 0.670 | 0.085 |
|  |  |  |  |  |  |  |  |  |  |  |
| Control Mean | 0.019 | 0.027 | 0.012 | 0.132 | 0.397 | 0.200 | 0.049 | 0.143 | 0.310 | 0.010 |
| Asthma Mean | 0.102 | 0.027 | 0.047 | 0.594 | 0.550 | 0.720 | 1.012 | 0.326 | 1.294 | 0.210 |
| Fold Change | 5.530 | 0.988 | 3.853 | 4.513 | 1.387 | 3.607 | 20.871 | 2.280 | 4.173 | 2.101 |
| P Value | 0.249 | 0.398 | 0.209 | 0.413 | 0.841 | 0.111 | 0.195 | 0.607 | 0.095 | 0.310 |
|  |  |  |  |  |  |  |  |  |  |  |

**S4: Genes with significant differential expression in asthmatic subjects compared to control subjects.** Analysis performed by sequencing mRNA isolated from the plasma samples taken from asthma subjects (n = 4) and control subjects (n = 5) and mapping the sequenced mRNA to the to the hg19 human genome using the Tuxedo protocol (Galaxy software). Differential gene expression was carried out using CuffDiff (Galaxy software) and genes with a log2 fold change greater than 2.0 and a Q value < 0.05 were determined to have significant differential expression

| **gene** | **Control Mean** | **Asthma Mean** | **Fold Change (log2)** | **Expression State** | **p_value** | **q_value** |
| --- | --- | --- | --- | --- | --- | --- |
| HLA-DQA1 | 0 | 38.842 | inf | Upregulated | 0.00005 | 0.002975 |
| IRF2 | 231.862 | 1.90169 | -6.92984 | Downregulated | 0.00185 | 0.046128 |
| VDR | 1.11565 | 24.3307 | 4.44682 | Upregulated | 0.0016 | 0.042721 |
| ABCF2 | 14.1914 | 0.36141 | -5.29524 | Downregulated | 0.0006 | 0.021645 |
| ACY3 | 13.5453 | 0 | -inf | Downregulated | 0.00005 | 0.002975 |
| ADAMTS18 | 4.84415 | 0 | -inf | Downregulated | 0.00005 | 0.002975 |
| ADHFE1 | 0 | 4.40631 | inf | Upregulated | 0.0002 | 0.00929 |
| AGTPBP1 | 2641.03 | 10.9345 | -7.91608 | Downregulated | 0.00075 | 0.02582 |
| AIP | 170.828 | 2.38999 | -6.1594 | Downregulated | 0.00185 | 0.046128 |
| AKAP12 | 29.4358 | 0.804642 | -5.19308 | Downregulated | 0.0017 | 0.044005 |
| ALX4 | 9.65369 | 0 | -inf | Downregulated | 0.00005 | 0.002975 |
| ALYREF | 33.614 | 0.682251 | -5.62261 | Downregulated | 0.00175 | 0.044787 |
| ANAPC4 | 64.985 | 1.63525 | -5.31253 | Downregulated | 0.002 | 0.048099 |
| ANKHD1,ANKHD1-EIF4EBP3,EIF4EBP3 | 33.1389 | 0.913791 | -5.18052 | Downregulated | 0.0002 | 0.00929 |
| ANKRD11 | 20.86 | 0.480761 | -5.43927 | Downregulated | 0.00015 | 0.007164 |
| ANKRD62P1-PARP4P3 | 17.4654 | 0 | -inf | Downregulated | 0.00005 | 0.002975 |
| ANO2 | 6.01042 | 0 | -inf | Downregulated | 0.00005 | 0.002975 |
| ARMC3 | 0 | 3.27624 | inf | Upregulated | 0.00005 | 0.002975 |
| ARV1 | 14.0641 | 0 | -inf | Downregulated | 0.00005 | 0.002975 |
| ASCC3 | 9.03307 | 0.512389 | -4.13991 | Downregulated | 0.00185 | 0.046128 |
| ASPH | 15.551 | 2.12998 | -2.8681 | Downregulated | 0.0011 | 0.03391 |
| B4GALT5 | 11.1549 | 0.320427 | -5.12154 | Downregulated | 0.0015 | 0.040855 |
| BPI | 0 | 2.22016 | inf | Upregulated | 0.00005 | 0.002975 |
| BRI3BP | 5.77652 | 0 | -inf | Downregulated | 0.00005 | 0.002975 |
| C10orf58 | 1100.39 | 1.23777 | -9.79605 | Downregulated | 0.00025 | 0.010799 |
| C15orf2 | 9.27968 | 0 | -inf | Downregulated | 0.00005 | 0.002975 |
| C15orf41 | 79.1979 | 0 | -inf | Downregulated | 0.00005 | 0.002975 |
| C16orf96 | 1.30855 | 0 | -inf | Downregulated | 0.00005 | 0.002975 |
| C17orf76-AS1,SNORD49B | 1093.04 | 33.3044 | -5.03649 | Downregulated | 0.00005 | 0.002975 |
| C19orf12 | 13.6132 | 0.198599 | -6.09901 | Downregulated | 0.00175 | 0.044787 |
| C20orf123 | 0 | 2.20453 | inf | Upregulated | 0.0001 | 0.005217 |
| C4orf48 | 3.29428 | 0 | -inf | Downregulated | 0.0012 | 0.035852 |
| CABP5 | 5.29506 | 122.071 | 4.52693 | Upregulated | 0.0017 | 0.044005 |
| CAPRIN1 | 393.033 | 10.253 | -5.26052 | Downregulated | 0.00195 | 0.047572 |
| CCDC75 | 5.79125 | 0 | -inf | Downregulated | 0.0001 | 0.005217 |
| CCDC85A | 25.2519 | 0 | -inf | Downregulated | 0.00005 | 0.002975 |
| CCDC85B | 209.821 | 1.87408 | -6.80684 | Downregulated | 0.001 | 0.031692 |
| CD300A | 71.4662 | 0 | -inf | Downregulated | 0.00005 | 0.002975 |
| CD46 | 154.097 | 5.32019 | -4.85622 | Downregulated | 0.0008 | 0.026859 |
| CD93 | 0 | 14.3366 | inf | Upregulated | 0.00025 | 0.010799 |
| CDCP2 | 2.39544 | 0 | -inf | Downregulated | 0.0001 | 0.005217 |
| CDH5 | 14.3453 | 0 | -inf | Downregulated | 0.00005 | 0.002975 |
| CEBPA | 2.3114 | 0 | -inf | Downregulated | 0.00015 | 0.007164 |
| CHD1L | 363.845 | 7.68883 | -5.56442 | Downregulated | 0.0009 | 0.029345 |
| CHMP1A | 898.053 | 10.7756 | -6.38096 | Downregulated | 0.0003 | 0.012559 |
| CKM | 0 | 3.70271 | inf | Upregulated | 0.00005 | 0.002975 |
| CLTB | 10.9768 | 241.021 | 4.45664 | Upregulated | 0.0017 | 0.044005 |
| CNTNAP3B | 68.4211 | 0 | -inf | Downregulated | 0.00005 | 0.002975 |
| CSF2RB | 6.86202 | 0 | -inf | Downregulated | 0.00005 | 0.002975 |
| CTSG | 9.81237 | 0 | -inf | Downregulated | 0.00005 | 0.002975 |
| CTSL1 | 69.2284 | 0.578126 | -6.90384 | Downregulated | 0.0008 | 0.026859 |
| CUL3 | 25.0839 | 0.317112 | -6.30563 | Downregulated | 0.0005 | 0.019051 |
| CYB5RL | 9.31978 | 0 | -inf | Downregulated | 0.00005 | 0.002975 |
| DCTN1 | 25.8084 | 1.60611 | -4.0062 | Downregulated | 0.00105 | 0.032968 |
| DCUN1D2 | 6.92677 | 0 | -inf | Downregulated | 0.00005 | 0.002975 |
| DOHH | 972.908 | 0 | -inf | Downregulated | 0.00005 | 0.002975 |
| DVL3 | 3.47278 | 0.166041 | -4.38648 | Downregulated | 0.00165 | 0.043542 |
| EBF2 | 0 | 65.1403 | inf | Upregulated | 0.0003 | 0.012559 |
| EID2B | 0 | 4.0054 | inf | Upregulated | 0.00005 | 0.002975 |
| EIF4G1 | 17.7869 | 1.88297 | -3.23973 | Downregulated | 0.00145 | 0.040138 |
| ELOF1 | 806.425 | 17.8618 | -5.49659 | Downregulated | 0.00045 | 0.017952 |
| EMILIN2 | 0 | 1.11829 | inf | Upregulated | 0.00005 | 0.002975 |
| ENOPH1 | 128.094 | 0.503077 | -7.99221 | Downregulated | 0.00205 | 0.048955 |
| F3 | 136.603 | 7.23521 | -4.23881 | Downregulated | 0.00055 | 0.020383 |
| FAM131C | 5.97135 | 0 | -inf | Downregulated | 0.00005 | 0.002975 |
| FAM183B | 9.24909 | 0 | -inf | Downregulated | 0.00005 | 0.002975 |
| FAM26F | 16.122 | 0 | -inf | Downregulated | 0.00005 | 0.002975 |
| FAM43A | 12.3554 | 0 | -inf | Downregulated | 0.00005 | 0.002975 |
| FBXL19 | 87.8135 | 0.890567 | -6.62357 | Downregulated | 0.00075 | 0.02582 |
| FBXO40 | 16.8548 | 0 | -inf | Downregulated | 0.00005 | 0.002975 |
| FETUB | 0 | 11.7485 | inf | Upregulated | 0.00005 | 0.002975 |
| FGF20 | 3.36049 | 0 | -inf | Downregulated | 0.00005 | 0.002975 |
| FGFBP2 | 5.32951 | 0 | -inf | Downregulated | 0.00005 | 0.002975 |
| FN1 | 240.158 | 33.3872 | -2.84662 | Downregulated | 0.00005 | 0.002975 |
| FUS | 101.743 | 5.78593 | -4.13624 | Downregulated | 0.0011 | 0.03391 |
| GABPB2 | 10.6607 | 0 | -inf | Downregulated | 0.00005 | 0.002975 |
| GBP4 | 0 | 12.9084 | inf | Upregulated | 0.00005 | 0.002975 |
| GDF7 | 1.19163 | 0 | -inf | Downregulated | 0.00005 | 0.002975 |
| GOLGA6L10 | 5.22453 | 0 | -inf | Downregulated | 0.00005 | 0.002975 |
| GOSR2 | 5.22701 | 97.5859 | 4.22261 | Upregulated | 0.002 | 0.048099 |
| GPC2 | 12.5745 | 0 | -inf | Downregulated | 0.00005 | 0.002975 |
| GPIHBP1 | 7.78948 | 0 | -inf | Downregulated | 0.00005 | 0.002975 |
| GPR108 | 8.48897 | 0.440074 | -4.26977 | Downregulated | 0.0014 | 0.039398 |
| GPR141 | 915.003 | 0 | -inf | Downregulated | 0.00015 | 0.007164 |
| GPR56 | 1.86976 | 98.5377 | 5.71975 | Upregulated | 0.0003 | 0.012559 |
| GRB10 | 1.37782 | 56.4596 | 5.35676 | Upregulated | 0.0005 | 0.019051 |
| GRK6 | 107.048 | 2.21174 | -5.59693 | Downregulated | 0.00025 | 0.010799 |
| GSTA1 | 0 | 25.5876 | inf | Upregulated | 0.00005 | 0.002975 |
| HBG2 | 0 | 8.79997 | inf | Upregulated | 0.0001 | 0.005217 |
| HBM | 0 | 7.92082 | inf | Upregulated | 0.0005 | 0.019051 |
| HDAC9 | 0.731644 | 52.1632 | 6.15575 | Upregulated | 0.0001 | 0.005217 |
| HERC2P3 | 2.27412 | 0 | -inf | Downregulated | 0.00005 | 0.002975 |
| HINT2 | 0 | 2.96934 | inf | Upregulated | 0.00015 | 0.007164 |
| HIST1H2AB | 0 | 15.1707 | inf | Upregulated | 0.00025 | 0.010799 |
| HIST1H2BI | 251.282 | 0 | -inf | Downregulated | 0.00045 | 0.017952 |
| HIST1H3C | 0 | 90.5782 | inf | Upregulated | 0.00005 | 0.002975 |
| HIST1H3E | 40.2474 | 0 | -inf | Downregulated | 0.00005 | 0.002975 |
| HIST1H3G | 1807.63 | 8.41281 | -7.7473 | Downregulated | 0.0012 | 0.035852 |
| HIST1H3I | 30.2331 | 0 | -inf | Downregulated | 0.00005 | 0.002975 |
| HIST1H4D | 2636.81 | 0 | -inf | Downregulated | 0.00005 | 0.002975 |
| HIST2H2AC | 6.23859 | 791.766 | 6.98771 | Upregulated | 0.00115 | 0.034818 |
| HIST3H2A | 0 | 13.2952 | inf | Upregulated | 0.00005 | 0.002975 |
| HLA-DRB5 | 6.09019 | 0 | -inf | Downregulated | 0.00005 | 0.002975 |
| HOTTIP | 0 | 28.228 | inf | Upregulated | 0.00005 | 0.002975 |
| HOXB4 | 6.12646 | 0 | -inf | Downregulated | 0.00005 | 0.002975 |
| HOXC10 | 26.4924 | 0 | -inf | Downregulated | 0.00005 | 0.002975 |
| HRC | 7.67678 | 0 | -inf | Downregulated | 0.00005 | 0.002975 |
| HSH2D | 56.2425 | 2.55331 | -4.46122 | Downregulated | 0.0019 | 0.046858 |
| HSP90AB3P | 169.495 | 1.84365 | -6.52254 | Downregulated | 0.00155 | 0.041715 |
| HUNK | 1.10728 | 0 | -inf | Downregulated | 0.00005 | 0.002975 |
| IBSP | 8.86449 | 0 | -inf | Downregulated | 0.00005 | 0.002975 |
| IDH3B | 121.794 | 0.585332 | -7.70097 | Downregulated | 0.0007 | 0.024598 |
| IKBKB | 33.2985 | 1.09014 | -4.93287 | Downregulated | 0.0006 | 0.021645 |
| IL4I1,NUP62 | 95.85 | 0.298023 | -8.32921 | Downregulated | 0.0013 | 0.037517 |
| IL7R | 95.0278 | 0 | -inf | Downregulated | 0.00005 | 0.002975 |
| IMPDH1 | 10.8144 | 212.562 | 4.29685 | Upregulated | 0.0009 | 0.029345 |
| INSIG1 | 136.506 | 6.80182 | -4.32691 | Downregulated | 0.001 | 0.031692 |
| IPO9 | 7.62404 | 0.078424 | -6.60311 | Downregulated | 0.00075 | 0.02582 |
| ITPKA | 0 | 1.83443 | inf | Upregulated | 0.00005 | 0.002975 |
| JAM2 | 0 | 21.0968 | inf | Upregulated | 0.00005 | 0.002975 |
| JMJD7,JMJD7-PLA2G4B,PLA2G4B | 44.1466 | 0.457344 | -6.59288 | Downregulated | 0.0016 | 0.042721 |
| KDM4DL | 0 | 1.54284 | inf | Upregulated | 0.0002 | 0.00929 |
| KIAA1211 | 0 | 10.4494 | inf | Upregulated | 0.00005 | 0.002975 |
| KIF26A | 10.373 | 0.189802 | -5.77219 | Downregulated | 0.0013 | 0.037517 |
| KLHL20 | 4464.31 | 5.4156 | -9.6871 | Downregulated | 0.00185 | 0.046128 |
| KLRF1 | 0 | 275.313 | inf | Upregulated | 0.00025 | 0.010799 |
| KRT9 | 2.07011 | 0 | -inf | Downregulated | 0.0001 | 0.005217 |
| LDHA | 441.515 | 18.4853 | -4.57801 | Downregulated | 0.00165 | 0.043542 |
| LGALS3 | 171.195 | 6.68305 | -4.67899 | Downregulated | 0.00055 | 0.020383 |
| LILRA1 | 11.1223 | 0 | -inf | Downregulated | 0.00005 | 0.002975 |
| LINC00085 | 15.0233 | 0 | -inf | Downregulated | 0.00005 | 0.002975 |
| LMO4 | 35.3109 | 0 | -inf | Downregulated | 0.0006 | 0.021645 |
| LOC100128239 | 2.69488 | 0 | -inf | Downregulated | 0.00005 | 0.002975 |
| LOC100499405 | 9.05866 | 0 | -inf | Downregulated | 0.00005 | 0.002975 |
| LOC100507003 | 0 | 6.79373 | inf | Upregulated | 0.00005 | 0.002975 |
| LOC100507632 | 0 | 2.66394 | inf | Upregulated | 0.0001 | 0.005217 |
| LOC399829 | 0 | 0.973263 | inf | Upregulated | 0.00005 | 0.002975 |
| LOC401127 | 5.4101 | 0 | -inf | Downregulated | 0.00005 | 0.002975 |
| LOC653653 | 0 | 30.0713 | inf | Upregulated | 0.00005 | 0.002975 |
| LOC730102 | 10.8399 | 0 | -inf | Downregulated | 0.00005 | 0.002975 |
| LRRD1 | 9.76312 | 0 | -inf | Downregulated | 0.0001 | 0.005217 |
| MAP7 | 5.38463 | 0.266441 | -4.33696 | Downregulated | 0.0001 | 0.005217 |
| MBD1 | 77.8778 | 0.422578 | -7.52585 | Downregulated | 0.00005 | 0.002975 |
| MCM3 | 30.3588 | 0.286844 | -6.7257 | Downregulated | 0.00025 | 0.010799 |
| MEG8 | 51.0706 | 0 | -inf | Downregulated | 0.00005 | 0.002975 |
| MIOX | 1.53897 | 0 | -inf | Downregulated | 0.00005 | 0.002975 |
| MPO | 0 | 112.127 | inf | Upregulated | 0.0008 | 0.026859 |
| MPRIP | 14.3712 | 0.415397 | -5.11255 | Downregulated | 0.001 | 0.031692 |
| MR1 | 1.07632 | 17.8916 | 4.0551 | Upregulated | 0.00045 | 0.017952 |
| MRPL54 | 6482.84 | 11.7281 | -9.11051 | Downregulated | 0.0015 | 0.040855 |
| MSH2 | 310.346 | 3.43451 | -6.49763 | Downregulated | 0.0005 | 0.019051 |
| MXRA7 | 9.57216 | 152.388 | 3.99276 | Upregulated | 0.00055 | 0.020383 |
| NADSYN1 | 437.838 | 4.64913 | -6.55729 | Downregulated | 0.0006 | 0.021645 |
| NCAN | 12.1701 | 0 | -inf | Downregulated | 0.0001 | 0.005217 |
| NCF1C | 307.061 | 4.56667 | -6.07124 | Downregulated | 0.0011 | 0.03391 |
| NCOA3 | 0.627287 | 11.2265 | 4.16164 | Upregulated | 0.00165 | 0.043542 |
| NDUFB6 | 23.8632 | 0 | -inf | Downregulated | 0.0021 | 0.049624 |
| NEK9 | 554.031 | 6.60108 | -6.39112 | Downregulated | 0.0021 | 0.049624 |
| NFXL1 | 17.8423 | 0 | -inf | Downregulated | 0.00005 | 0.002975 |
| NHLRC4 | 0 | 170.224 | inf | Upregulated | 0.00065 | 0.023202 |
| NID2 | 0 | 14.7488 | inf | Upregulated | 0.00005 | 0.002975 |
| NINJ2 | 0 | 7.06839 | inf | Upregulated | 0.00005 | 0.002975 |
| NKAPL | 11.1102 | 0 | -inf | Downregulated | 0.00005 | 0.002975 |
| NLRP6 | 0 | 0.859184 | inf | Upregulated | 0.00005 | 0.002975 |
| NPEPL1,STX16 | 126.825 | 9.62575 | -3.71979 | Downregulated | 0.0015 | 0.040855 |
| NRP1 | 0.92425 | 18.8945 | 4.35354 | Upregulated | 0.00025 | 0.010799 |
| NTS | 0 | 13.7899 | inf | Upregulated | 0.0001 | 0.005217 |
| OAS2 | 391.459 | 5.09555 | -6.26348 | Downregulated | 0.0005 | 0.019051 |
| OLFM2 | 21.3072 | 0 | -inf | Downregulated | 0.00025 | 0.010799 |
| PAK4 | 821.348 | 1.10351 | -9.53974 | Downregulated | 0.0014 | 0.039398 |
| PDE12 | 47.4721 | 1.76684 | -4.74784 | Downregulated | 0.00195 | 0.047572 |
| PDE4A | 2.36207 | 0 | -inf | Downregulated | 0.00005 | 0.002975 |
| PDGFRL | 0 | 148.208 | inf | Upregulated | 0.00055 | 0.020383 |
| PDK2 | 200.007 | 2.43951 | -6.35731 | Downregulated | 0.0013 | 0.037517 |
| PDLIM5 | 5997.3 | 84.1691 | -6.15488 | Downregulated | 0.00005 | 0.002975 |
| PFDN5 | 4816.4 | 36.2157 | -7.05519 | Downregulated | 0.00035 | 0.014474 |
| PIEZO2 | 4.18421 | 0 | -inf | Downregulated | 0.00005 | 0.002975 |
| PKHD1L1 | 0 | 27.9196 | inf | Upregulated | 0.00015 | 0.007164 |
| PLEKHG1 | 175.393 | 1.89737 | -6.53045 | Downregulated | 0.0017 | 0.044005 |
| PLEKHG5,TNFRSF25 | 13.8957 | 1.55313 | -3.16139 | Downregulated | 0.0009 | 0.029345 |
| PML | 0.948462 | 178.238 | 7.554 | Upregulated | 0.00015 | 0.007164 |
| PMM2 | 129.114 | 2.49069 | -5.69596 | Downregulated | 0.0015 | 0.040855 |
| PNMA2 | 16.2757 | 0 | -inf | Downregulated | 0.00005 | 0.002975 |
| PNMT | 4.37229 | 0 | -inf | Downregulated | 0.00005 | 0.002975 |
| PNPLA6 | 35.5727 | 0.369179 | -6.59031 | Downregulated | 0.0006 | 0.021645 |
| POLRMT | 9.4577 | 0.36157 | -4.70914 | Downregulated | 0.0018 | 0.045893 |
| PPEF2 | 0 | 18.0939 | inf | Upregulated | 0.00005 | 0.002975 |
| PPM1N | 0 | 2.65807 | inf | Upregulated | 0.00005 | 0.002975 |
| PPP1CA | 2049.79 | 7.9759 | -8.00561 | Downregulated | 0.0013 | 0.037517 |
| PPP1R3G | 14.9547 | 0 | -inf | Downregulated | 0.00005 | 0.002975 |
| PPP2R5C | 75.7852 | 1.35599 | -5.8045 | Downregulated | 0.00115 | 0.034818 |
| PRAM1 | 0 | 3.05743 | inf | Upregulated | 0.0001 | 0.005217 |
| PRKAA1 | 97.1062 | 1.68592 | -5.84796 | Downregulated | 0.00095 | 0.030535 |
| PROCA1 | 7.46774 | 0 | -inf | Downregulated | 0.00005 | 0.002975 |
| PROL1 | 27.6132 | 0 | -inf | Downregulated | 0.00005 | 0.002975 |
| PROSC | 3.60962 | 98.0712 | 4.76391 | Upregulated | 0.0014 | 0.039398 |
| PRR12 | 213.417 | 0.500231 | -8.73687 | Downregulated | 0.00145 | 0.040138 |
| PSMB11 | 4.89107 | 0 | -inf | Downregulated | 0.00005 | 0.002975 |
| PSMD9 | 192.358 | 1.2033 | -7.32065 | Downregulated | 0.0005 | 0.019051 |
| PTPN23 | 0.217047 | 6.74238 | 4.95718 | Upregulated | 0.00115 | 0.034818 |
| PTRH2 | 87.7907 | 0 | -inf | Downregulated | 0.00005 | 0.002975 |
| PTS | 9.20291 | 0 | -inf | Downregulated | 0.0009 | 0.029345 |
| QSOX1 | 7.57226 | 0.559299 | -3.75903 | Downregulated | 0.00105 | 0.032968 |
| RAB18 | 2.69093 | 143.96 | 5.74142 | Upregulated | 0.00125 | 0.037182 |
| RAB3IL1 | 15.1233 | 0 | -inf | Downregulated | 0.00005 | 0.002975 |
| RAB6B | 0 | 8.90346 | inf | Upregulated | 0.00015 | 0.007164 |
| RAD18 | 0 | 2.923 | inf | Upregulated | 0.0001 | 0.005217 |
| RASGEF1B | 0 | 2.80288 | inf | Upregulated | 0.00045 | 0.017952 |
| RBM10 | 11.7999 | 0.116634 | -6.66064 | Downregulated | 0.00015 | 0.007164 |
| RBM14,RBM14-RBM4,RBM4 | 3.40911 | 169.88 | 5.63898 | Upregulated | 0.0012 | 0.035852 |
| RBM39 | 8.85084 | 152.169 | 4.10372 | Upregulated | 0.00185 | 0.046128 |
| RCSD1 | 143.939 | 2.56999 | -5.80754 | Downregulated | 0.0007 | 0.024598 |
| RFNG | 67.4227 | 0.354253 | -7.57231 | Downregulated | 0.00155 | 0.041715 |
| RNF149 | 138.204 | 5.88687 | -4.55316 | Downregulated | 0.00145 | 0.040138 |
| ROCK1P1 | 0 | 9.2626 | inf | Upregulated | 0.0002 | 0.00929 |
| RPL34 | 1861.93 | 30.2819 | -5.9422 | Downregulated | 0.0005 | 0.019051 |
| RPLP0P2 | 2.2382 | 0 | -inf | Downregulated | 0.00005 | 0.002975 |
| RPS5 | 29863.9 | 4240.63 | -2.81606 | Downregulated | 0.00065 | 0.023202 |
| RRN3P1 | 0 | 10.7453 | inf | Upregulated | 0.0001 | 0.005217 |
| SDF2L1 | 226.184 | 8.69692 | -4.70085 | Downregulated | 0.0021 | 0.049624 |
| SEMA3G | 7.03633 | 0 | -inf | Downregulated | 0.00005 | 0.002975 |
| SENP1 | 77.9831 | 0.994579 | -6.29293 | Downregulated | 0.002 | 0.048099 |
| SH2D1B | 0 | 2.2877 | inf | Upregulated | 0.0013 | 0.037517 |
| SHANK3 | 258.046 | 1.17076 | -7.78404 | Downregulated | 0.002 | 0.048099 |
| SHC1 | 303.85 | 4.55052 | -6.06118 | Downregulated | 0.00015 | 0.007164 |
| SKIL | 3.5632 | 249.498 | 6.12971 | Upregulated | 0.00195 | 0.047572 |
| SLAMF1 | 0 | 24.4321 | inf | Upregulated | 0.00005 | 0.002975 |
| SLC2A3 | 2562.68 | 4.50521 | -9.15185 | Downregulated | 0.00145 | 0.040138 |
| SLC39A13 | 37.0797 | 0.190603 | -7.60392 | Downregulated | 0.00135 | 0.038632 |
| SLC41A2 | 0 | 5.46035 | inf | Upregulated | 0.0003 | 0.012559 |
| SLC43A2 | 599.19 | 0.988295 | -9.24386 | Downregulated | 0.00095 | 0.030535 |
| SLCO4C1 | 0 | 26.1018 | inf | Upregulated | 0.00005 | 0.002975 |
| SMTN | 1.08151 | 14.7549 | 3.77008 | Upregulated | 0.0013 | 0.037517 |
| SOX18 | 0 | 3.09935 | inf | Upregulated | 0.00015 | 0.007164 |
| SPC24 | 175.741 | 0 | -inf | Downregulated | 0.00015 | 0.007164 |
| SPSB3 | 223.945 | 8.42024 | -4.73314 | Downregulated | 0.00175 | 0.044787 |
| SSTR1 | 1.88179 | 0 | -inf | Downregulated | 0.00005 | 0.002975 |
| ST3GAL4 | 37.7156 | 0.728955 | -5.69319 | Downregulated | 0.00055 | 0.020383 |
| ST8SIA2 | 1.93328 | 0 | -inf | Downregulated | 0.00005 | 0.002975 |
| ST8SIA3 | 7.9204 | 0 | -inf | Downregulated | 0.00005 | 0.002975 |
| STAG2 | 1.2453 | 76.6784 | 5.94425 | Upregulated | 0.0009 | 0.029345 |
| STRN4 | 1.01732 | 102.211 | 6.65063 | Upregulated | 0.0007 | 0.024598 |
| STYX | 417.178 | 5.40866 | -6.26925 | Downregulated | 0.0008 | 0.026859 |
| SUV420H2 | 2.51161 | 0 | -inf | Downregulated | 0.00005 | 0.002975 |
| SYNDIG1L | 0 | 4.40342 | inf | Upregulated | 0.00005 | 0.002975 |
| TAOK2 | 15.0671 | 0.142532 | -6.72398 | Downregulated | 0.00135 | 0.038632 |
| TARSL2 | 3.76791 | 0 | -inf | Downregulated | 0.00005 | 0.002975 |
| TBX15 | 8.60566 | 0 | -inf | Downregulated | 0.00005 | 0.002975 |
| TCP11L2 | 363.919 | 5.85202 | -5.95854 | Downregulated | 0.00045 | 0.017952 |
| TEKT2 | 2.95065 | 0 | -inf | Downregulated | 0.00005 | 0.002975 |
| TIGIT | 0 | 9.41916 | inf | Upregulated | 0.00005 | 0.002975 |
| TIMELESS | 60.8055 | 0.980945 | -5.95389 | Downregulated | 0.00085 | 0.028398 |
| TLR1 | 0 | 104.195 | inf | Upregulated | 0.00035 | 0.014474 |
| TMC1 | 6.10844 | 0 | -inf | Downregulated | 0.00005 | 0.002975 |
| TMCC1 | 15.401 | 0.238694 | -6.01171 | Downregulated | 0.00025 | 0.010799 |
| TMEM151A | 0 | 3.256 | inf | Upregulated | 0.00005 | 0.002975 |
| TMSB15A | 0 | 34.918 | inf | Upregulated | 0.00015 | 0.007164 |
| TNFRSF13C | 0 | 12.7534 | inf | Upregulated | 0.0001 | 0.005217 |
| TOMM22 | 772.966 | 23.9815 | -5.01041 | Downregulated | 0.00115 | 0.034818 |
| TOP1MT | 0.344555 | 59.0342 | 7.42067 | Upregulated | 0.00045 | 0.017952 |
| TPRKB | 0 | 29.8617 | inf | Upregulated | 0.00025 | 0.010799 |
| TRADD | 215.12 | 0.796014 | -8.07813 | Downregulated | 0.00075 | 0.02582 |
| TRAPPC6A | 12.5852 | 0 | -inf | Downregulated | 0.00005 | 0.002975 |
| TRIOBP | 182.005 | 1.40604 | -7.01619 | Downregulated | 0.0014 | 0.039398 |
| TRNT1 | 0 | 21.9223 | inf | Upregulated | 0.00005 | 0.002975 |
| TSPYL5 | 18.9517 | 0 | -inf | Downregulated | 0.00005 | 0.002975 |
| TTBK1 | 1.24198 | 0 | -inf | Downregulated | 0.00005 | 0.002975 |
| TXK | 4.01629 | 0 | -inf | Downregulated | 0.00005 | 0.002975 |
| UBE2G1 | 2341.68 | 15.7889 | -7.21249 | Downregulated | 0.0013 | 0.037517 |
| UBE2NL | 0 | 4.49875 | inf | Upregulated | 0.0008 | 0.026859 |
| UBXN10 | 0 | 17.0071 | inf | Upregulated | 0.00005 | 0.002975 |
| UNC13C | 0 | 3.90667 | inf | Upregulated | 0.00005 | 0.002975 |
| UQCRB | 0.895524 | 104.973 | 6.87307 | Upregulated | 0.00155 | 0.041715 |
| VPS13C | 9.57266 | 0.383193 | -4.64278 | Downregulated | 0.00205 | 0.048955 |
| VPS8 | 58.8726 | 2.52771 | -4.5417 | Downregulated | 0.0003 | 0.012559 |
| WDR87 | 6.16026 | 0 | -inf | Downregulated | 0.0005 | 0.019051 |
| YPEL4 | 0 | 97.0244 | inf | Upregulated | 0.00025 | 0.010799 |
| ZCCHC12 | 14.713 | 0 | -inf | Downregulated | 0.00005 | 0.002975 |
| ZFAND6 | 33.1129 | 877.521 | 4.72797 | Upregulated | 0.0019 | 0.046858 |
| ZIC1 | 3.52814 | 0 | -inf | Downregulated | 0.00005 | 0.002975 |
| ZNF101 | 0 | 2.61671 | inf | Upregulated | 0.00095 | 0.030535 |
| ZNF335 | 18.312 | 0.104569 | -7.45218 | Downregulated | 0.0011 | 0.03391 |
| ZNF584 | 10.6349 | 0 | -inf | Downregulated | 0.00005 | 0.002975 |
| ZNF710 | 132.55 | 0.804927 | -7.36346 | Downregulated | 0.00185 | 0.046128 |
| ZNF876P | 0 | 56.8231 | inf | Upregulated | 0.0001 | 0.005217 |
| ZNRF2 | 18.1662 | 0 | -inf | Downregulated | 0.0019 | 0.046858 |
| ZSWIM3 | 4.34792 | 0 | -inf | Downregulated | 0.0017 | 0.044005 |

**S5: miRNA with significant differential expression in asthmatic subjects compared to control subjects.** Analysis performed by sequencing miRNA isolated from plasma samples taken from asthmatic subjects (n = 5) and control subjects (n = 5) and mapping the miRNA reads to the human miRbase Version 21 using sRNAtoolbox. miRNA was quantified using Fragments Per Kilobase of transcript per Million mapped (FPKM) reads, and differential expression was determined using the edgeR program (Bioconductor software). Significant expression was defined as having a log fold change greater than 2.0 and a false rate of discovery (FDR) adjusted P value < 0.05.

| **miRNA** | **Control Mean (FPKM Score)** | **Asthma Mean (FPKM Score)** | **log Fold Change** | **Expression State** | **P value** | **FDR** |
| --- | --- | --- | --- | --- | --- | --- |
| hsa-miR-3928-3p | 0.236174 | 57.33876 | 7.158321 | Upregulated | 0.000233 | 0.033654 |
| hsa-miR-6772-3p | 1.055122 | 35.72978 | 4.602044 | Upregulated | 0.000482 | 0.033654 |
| hsa-miR-369-5p | 1.24549 | 59.39359 | 5.177801 | Upregulated | 0.000705 | 0.033654 |
| hsa-miR-326 | 7.2467 | 226.1252 | 4.749626 | Upregulated | 0.000816 | 0.033654 |
| hsa-miR-151a-3p | 27096.79 | 275400.1 | 3.345161 | Upregulated | 0.001552 | 0.036926 |
| hsa-miR-24-3p | 710.5873 | 12008.67 | 4.079512 | Upregulated | 0.001763 | 0.036926 |
| hsa-miR-548e-3p | 3.094352 | 59.72168 | 4.142479 | Upregulated | 0.001924 | 0.036926 |
| hsa-miR-1468-5p | 11.40445 | 153.5177 | 3.586362 | Upregulated | 0.002141 | 0.036926 |
| hsa-miR-493-3p | 10.36243 | 240.2131 | 4.359833 | Upregulated | 0.00219 | 0.036926 |
| hsa-miR-148a-3p | 11926.8 | 195898.3 | 4.037891 | Upregulated | 0.002238 | 0.036926 |
| hsa-miR-654-5p | 31.18251 | 722.2062 | 4.434117 | Upregulated | 0.002642 | 0.037494 |
| hsa-miR-382-5p | 1064.709 | 15861.82 | 3.896799 | Upregulated | 0.002727 | 0.037494 |
| hsa-miR-744-5p | 692.3024 | 4701.145 | 2.752171 | Upregulated | 0.003703 | 0.047004 |

**S6: Bacterial alpha diversity present in the plasma samples**. Alpha diversity was measured using rarefied OTU tables generated from 16S rRNA sequencing data from plasma samples collected from asthma subjects (n =5) and control subjects (n = 5). Shannon diversity index scores were generated from OTU tables in order to measure the richness of the plasma sample and evenness of bacterial taxa present in the sample. Chao1 index scores were measured to determine the predicted number of bacterial taxa present in the plasma samples by extrapolating out the number of rare organisms that may not have been detected due to under-sampling.

| **Sample ID** | **Number of Reads** | **Chao1 Index Score** | **Shannon Index Score** |
| --- | --- | --- | --- |
| Control_1 | 20367 | 357.00 | 2.17 |
| Control_2 | 34243 | 423.75 | 2.87 |
| Control_3 | 19680 | 402.14 | 2.27 |
| Control_4 | 10929 | 349.00 | 2.32 |
| Control_5 | 26469 | 444.58 | 2.83 |
| Asthma_1 | 36136 | 359.97 | 3.04 |
| Asthma_2 | 19459 | 358.29 | 3.03 |
| Asthma_3 | 35213 | 302.64 | 2.22 |
| Asthma_4 | 17155 | 409.00 | 3.02 |
| Asthma_5 | 27413 | 498.41 | 3.75 |
